# Oocytes maintain ROS-free mitochondrial metabolism by suppressing complex I

**DOI:** 10.1038/s41586-022-04979-5

**Published:** 2022-07-20

**Authors:** Aida Rodríguez-Nuevo, Ariadna Torres-Sanchez, Juan M. Duran, Cristian De Guirior, Maria Angeles Martínez-Zamora, Elvan Böke

**Affiliations:** 1 Centre for Genomic Regulation (CRG), The Barcelona Institute of Science and Technology, Barcelona, Spain; 2 Gynaecology Department, Institute Clinic of Gynaecology, Obstetrics and Neonatology, Hospital Clinic, Barcelona, Barcelona, Spain; 3 Faculty of Medicine, University of Barcelona, Barcelona, Spain; 4 Institut d’Investigacions Biomèdiques August Pi i Sunyer (IDIBAPS), Barcelona, Spain; 5 Universitat Pompeu Fabra (UPF), Barcelona, Spain

**Keywords:** Mitochondria, Oogenesis, Infertility

## Abstract

Oocytes form before birth and remain viable for several decades before fertilization^1^. Although poor oocyte quality accounts for most female fertility problems, little is known about how oocytes maintain cellular fitness, or why their quality eventually declines with age^2^. Reactive oxygen species (ROS) produced as by-products of mitochondrial activity are associated with lower rates of fertilization and embryo survival^3–5^. Yet, how healthy oocytes balance essential mitochondrial activity with the production of ROS is unknown. Here we show that oocytes evade ROS by remodelling the mitochondrial electron transport chain through elimination of complex I. Combining live-cell imaging and proteomics in human and *Xenopus* oocytes, we find that early oocytes exhibit greatly reduced levels of complex I. This is accompanied by a highly active mitochondrial unfolded protein response, which is indicative of an imbalanced electron transport chain. Biochemical and functional assays confirm that complex I is neither assembled nor active in early oocytes. Thus, we report a physiological cell type without complex I in animals. Our findings also clarify why patients with complex-I-related hereditary mitochondrial diseases do not experience subfertility. Complex I suppression represents an evolutionarily conserved strategy that allows longevity while maintaining biological activity in long-lived oocytes.

## Abstract (web summary)

Oocytes prevent the production of reactive oxygen species by remodelling the mitochondrial electron transport chain through elimination of complex I, a strategy that enables their long-term viability.

## Main

Human primordial oocytes are formed during fetal development and remain dormant in the ovary for up to 50 years. Despite a long period of dormancy, oocytes retain the ability to give rise to a new organism after fertilization. Decline in oocyte fitness is a key contributor to infertility with age^2^. However, little is known about how oocytes maintain cellular fitness for decades to preserve their developmental potential, complicating efforts to understand the declining oocyte quality in ageing women.

Oocytes remain metabolically active during dormancy^6,7^, and thus must maintain mitochondrial activity for biosynthesis of essential biomolecules^8^. Yet, mitochondria are a major source of ROS, generating them as by-products of mitochondrial oxidative metabolism. Although ROS can function as signalling molecules^9^, at high concentrations ROS promote DNA mutagenesis and are cytotoxic. Indeed, ROS levels are linked to apoptosis and reduced developmental competence in oocytes and embryos^3–5^. However, the mechanisms by which oocytes maintain this delicate balance between mitochondrial activity and ROS production have remained elusive.

## Mitochondrial ROS in early oocytes

**Extended Data Fig. 1:**
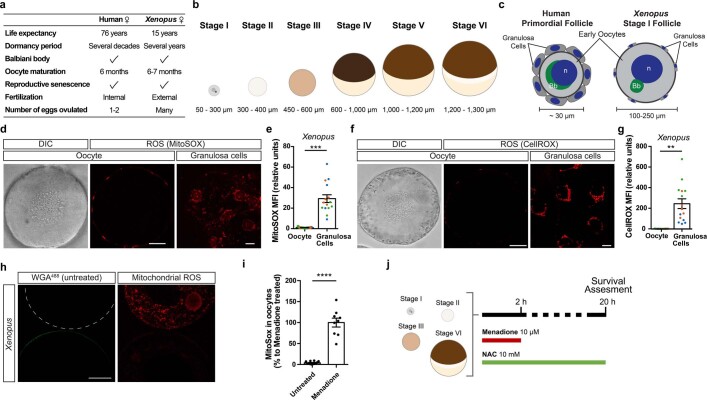
Undetectable levels of ROS in the early oocytes. **a**, A comparison between select reproductive traits of humans and *Xenopus laevis*, which live on average 76 years^59^ and 15 years in captivity^60^, respectively. *Xenopus* oocytes have long been used in reproduction studies^61,62^, in part because they are more accessible and share many conserved features with human oocytes, such as (1) a long dormancy period: *Xenopus* oocytes arrest at late-stage I for several years. Moreover, a large population of early oocytes is maintained in adult female ovaries throughout most of its life-time, suggesting the presence of an oocyte reserve similar to humans^63–65^. (2) A similar duration of the maturation period from early oocytes to mature eggs. (3) A measurable decline in fertility with age^60,66,67^. (4) A cytoplasmic distribution and activity of organelles similar to humans, including a Balbiani body found in the ooplasm of both species^6,68,69^. On the other hand, humans and *Xenopus* differ in their modes of fertilization: humans undergo internal fertilization, while *Xenopus* fertilization takes place externally. This important difference affects several features related to fertilization: *Xenopus* lay many eggs, each with considerable internal nutrient reserves for survival outside of the body, whereas humans ovulate only 1-2 eggs per cycle with little internal nutrient reserves^70–72^. **b**, A schematic of *Xenopus laevis* oogenesis according to^42^. Oogenesis in *Xenopus* is divided into six stages based on the morphology of the developing oocytes: oocytes are transparent and measure 50-300 microns in stage I. Oocytes grow and gradually accumulate pigments and yolk to become opaque and measure more than 1 mm in stage VI, when they are ready to be ovulated. **c**, Schematic representation of human and *Xenopus* early oocytes with attached granulosa cells. Nuclei (n) are depicted in blue and Balbiani bodies (Bb) in green. Note that *Xenopus* early oocytes are so large that their granulosa cells are visible only as small puncta on the periphery of the oocyte in the same magnification. **d**, **f**, Live-cell imaging of *Xenopus* early (stage I) oocytes with attached granulosa cells with MitoSOX (**d**), and CellROX (**f**) to detect their ROS levels. Granulosa cells were imaged in the basal plane of the oocyte. DIC, differential interference contrast. Scale bars: 50 µm and 10 µm for oocytes and granulosa cells, respectively. **e**, **g**, Quantification of MitoSOX (**e**) and CellROX (**g**) probes inside oocytes and in granulosa cells (n=3; biological replicates shown in colours). The data represent the mean ± s.e.m. ***P = 4.298 × 10^−8^ and **P = 1.86 × 10^−5^ using two-sided Student’s *t*-test. **h**, Live-cell imaging of ROS in early oocytes untreated or treated with menadione 10 µM for 2h. Untreated oocytes were incubated with Wheat Germ Agglutinin (WGA)^488^ (green) to mark the plasma membrane, then combined with treated oocytes in the same dish and labelled with MitoSOX. Scale bar: 50 µm. **i**, Quantification of MitoSOX in oocytes at the 4h timepoint (untreated or treated with 10 µM Menadione for 2h followed by 2h wash). The data represent the mean ± s.e.m, n=3 biological replicates, at least 3 oocytes were quantified per replicate ****P = 2.21 × 10^−9^ using two-sided Student’s *t*-test. **j**, Experimental design for the assessment of survival upon mild ROS production. Freshly isolated early (stage I), maturing (stage II and III), and late-stage (stage VI) oocytes of were treated with 10 µM menadione in the presence or in the absence of 10 mM *N*-acetyl cysteine (NAC). After 2h, menadione was removed and NAC was maintained for an overnight, when survival was determined. Source data

Early human oocytes can be accessed only through invasive surgery into ovaries. Therefore, biochemical investigations into oocyte biology have historically been hindered by severe sample limitations. As a consequence, mitochondrial activity in primordial oocytes remains largely unstudied. Here we overcome challenges imposed by human oocytes by utilizing an improved human oocyte isolation protocol recently developed in our laboratory^6^, which we combine with a comparative evolutionary approach using more readily available *Xenopus* stage I oocytes (both referred to as early oocytes hereafter; Extended Data Fig. 1a,b). This approach allowed us to generate hypotheses using multi-species or *Xenopus*-alone analyses, and subsequently test those hypotheses in human oocytes.

**Fig. 1:**
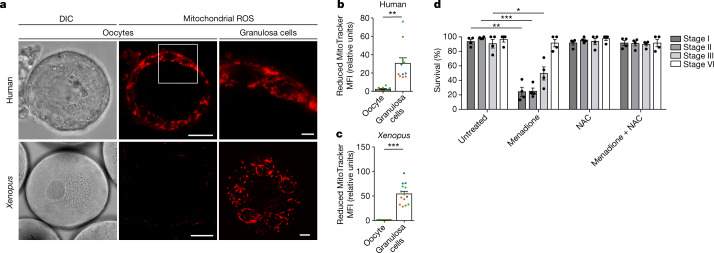
Early oocytes have undetectable levels of ROS. **a**, Live-cell imaging of human and *Xenopus* early oocytes, both with attached granulosa cells. The ROS level was measured using MitoTracker Red CM-H_2_XRos (H2X), a reduced mitochondrial dye that does not fluoresce until it is oxidized by ROS. The boxed area is magnified in the top right image. *Xenopus* granulosa cells were imaged at the basal plane of the oocyte. DIC, differential interference contrast. Scale bars, 15 µm (human oocytes), 50 µm (*Xenopus* oocytes), 3 µm (human granulosa cells) and 10 µm (*Xenopus* granulosa cells). **b**,**c**, Quantification of the mean fluorescence intensity (MFI) of H2X in the oocyte and in the population of granulosa cells surrounding the equatorial plane of the oocyte for human (**b**) and *Xenopus* (**c**) oocytes. The data represent the mean ± s.e.m. of three biological replicates, shown in different colours. **P = 0.0001 and ***P = 4.13 × 10^−11^ using a two-sided Student’s *t*-test. **d**, Overnight survival of oocytes at the indicated stages of oogenesis after treatment with menadione, *N*-acetyl cysteine (NAC) or the combination of both (see Extended Data Fig. 1j for experimental design). At least ten oocytes were incubated per condition. The data represent the mean ± s.e.m. across four biological replicates. *P = 1.94 × 10^−9^, **P = 3.77 × 10^−18^ and ***P = 2.37 × 10^−19^ compared with the untreated condition using a two-sided Student’s *t*-test with Šidák–Bonferroni-adjusted *P* values for multiple comparisons. Source data

We began our studies by imaging live early human and *Xenopus* oocytes labelled with various mitochondrial probes that quantify ROS levels. Neither *Xenopus* nor human early oocytes showed any detectable ROS signal, whereas mitochondria in somatic granulosa cells surrounding the oocytes exhibited ROS and served as positive controls (Fig. 1a–c and Extended Data Fig. 1c–g). ROS induction in oocytes also served as a positive control for live ROS probes (Extended Data Fig. 1h,i).

To distinguish between the possibilities that low ROS probe levels resulted from low ROS production or, alternatively, a high scavenging capacity to eliminate ROS, we treated *Xenopus* oocytes with menadione and assessed their survival (Extended Data Fig. 1j). Mild treatment with menadione promotes the formation of ROS (ref. ^10^) but does not affect survival negatively in cell lines and fruit flies^11,12^. However, most early oocytes (78.3%) died when they were left to recover overnight after menadione treatment, in contrast to what was observed for late-stage oocytes (Fig. 1d and Extended Data Fig. 1j). Treatment with an antioxidant that quenches ROS was able to rescue oocyte survival (Fig. 1d). These results indicate that evasion of ROS damage in oocytes involves tight control of ROS generation, rather than a higher scavenging capacity of oocytes against ROS.

## Mitochondrial respiration in oocytes

**Fig. 2:**
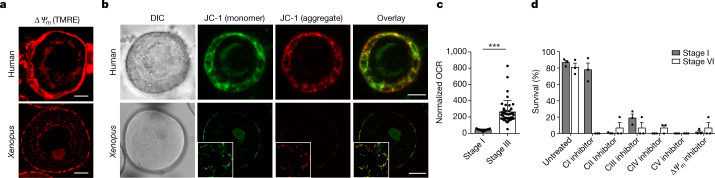
OXPHOS is low, but essential, in early oocytes. **a**,**b**, Live-cell imaging of human and *Xenopus* early oocytes with attached granulosa cells labelled with tetramethylrhodamine ethyl ester perchlorate (TMRE) to detect mitochondrial membrane potential (Δ*Ψ*_m_; **a**) and JC-1, a membrane potential sensitive binary dye (**b**). Green JC-1 fluorescence is a sign of low membrane potential; red fluorescence indicates JC-1 aggregation inside mitochondria, and thus high membrane potential. The insets in the *Xenopus* images show granulosa cells imaged in the basal plane of the oocyte. DIC, differential interference contrast. Scale bars, 10 µm (human oocytes) and 50 µm (*Xenopus* oocytes). Representative images are shown (see Extended Data Fig. 2 for quantification of independent experiments). **c**, The basal oxygen consumption rate in early (stage I) and growing (stage III) *Xenopus* oocytes, normalized for total protein per sample (*n* = 17 for stage I and *n* = 43 for stage III). The data represent mean ± s.e.m. ***P = 2.98 × 10^−8^ using a two-sided Student’s *t*-test. **d**, Overnight survival of early (stage I) and late (stage VI) oocytes after treatments with mitochondrial poisons: complex I (CI) to V (CV) inhibitors and an ionophore (5 µM rotenone, 50 mM malonic acid, 5 µM antimycin A, 50 mM KCN, 200 µM *N*,*N*′-dicyclohexylcarbodiimide (DCCD) or 30 µM carbonyl cyanide *m*-chlorophenyl hydrazone (CCCP), respectively). At least 50 early and 10 late-stage oocytes were incubated per condition. Δ*Ψ*_m_, mitochondrial membrane potential. The data represent the mean ± s.e.m. across three biological replicates. Source data

**Extended Data Fig. 2:**
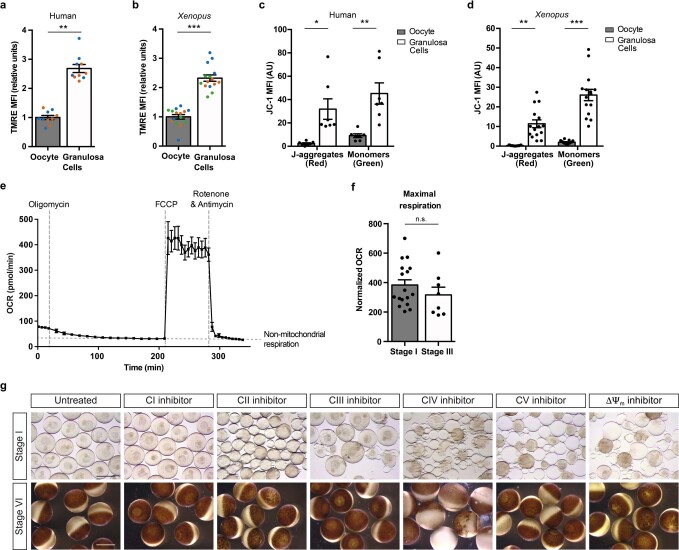
Oxidative phosphorylation is essential in early oocytes. **a**, **b**, Quantification of the mean fluorescence intensity (MFI) of TMRE in the oocyte and in the population of granulosa cells surrounding the equatorial plane of the oocyte for human (**a**) and *Xenopus* oocytes (**b**). The data represent the mean ± s.e.m. **P = 2.83 × 10^−9^ n=2 biological replicates in human; and ***P = 1.09 × 10^−10^ n=3 in *Xenopus;* using two-sided Student’s *t*-test. Replicates shown in colours. **c**, **d**, Quantification of the mean fluorescence intensity (MFI) of JC-1 aggregates (red) and monomers (green) in the oocyte and in the population of granulosa cells surrounding the equatorial plane of the oocyte for human (**c**) and *Xenopus* oocytes (**d**). We could not detect any red fluorescence in oocytes; an additional 2-hour incubation with JC-1 did not lead to detection of red fluorescence inside the oocyte either. The data represent the mean ± s.e.m. *P = 0.005 and **P = 0.002 in human, n=4; and **P = 5.67 × 10^−6^ and ***P = 4.42 × 10^−9^, n=3 in *Xenopus;* using two-sided Student’s *t*-test with Šidák-Bonferroni-adjusted *P* values for multiple comparisons. **e**, Oxygen consumption rate (OCR) as assessed by a seahorse analyser (XFe96) in early (stage I) *Xenopus* oocytes (mean ± SEM, n = 9). **f**, Maximal oxygen consumption rate in early (stage I) and growing (stage III) *Xenopus* oocytes, normalized for total protein/sample (n=17 for stage I and n=8 for stage III). The data represent the mean ± s.e.m. n.s. not significant (*P* = 0.299) using two-sided Student’s t-test. **g**, Representative images of *Xenopus* oocytes after an overnight treatment with mitochondrial poisons (5 µM Rotenone, 50 mM malonic acid, 5 µM antimycin A, 50 mM KCN, 200 µM DCCD or 30 µM CCCP). Upper panel shows early (stage I) oocytes and bottom panel displays late (stage VI) oocytes. Cell death can be recognized in early (stage I) oocytes by loss of plasma membrane integrity and/or loss of nucleus; and in late (stage VI) oocytes by development of a mottling pattern in the pigmentation of the animal pole (see main Fig. 2 for quantifications of three independent experiments). Scale bars: 250 µm and 1 mm for stage I and stage VI oocytes, respectively. Source data

Using dyes that sense membrane potential (tetramethylrhodamine ethyl ester perchlorate (TMRE) and the cyanine dye JC-1), we found that mitochondria in human and *Xenopus* early oocytes exhibit lower membrane potentials compared to those of neighbouring granulosa cells, which served as positive controls (Fig. 2a,b and Extended Data Fig. 2a–d). Undetectable ROS levels and low membrane potential suggest that the mitochondrial electron transport chain (ETC) activity in early oocytes is either low or absent. To differentiate between these two possibilities, we measured respiration rate in *Xenopus* oocytes. Early oocytes stripped of granulosa cells exhibited a low basal respiration rate but a similar maximal respiration rate compared to those of growing oocytes (Fig. 2c and Extended Data Fig. 2e,f). This respiration was efficiently coupled to ATP synthesis, resulting in an undetectable proton leak (Extended Data Fig. 2e). Therefore, we conclude that mitochondria in early oocytes have a functional ETC, with low activity.

To assess the importance of individual complexes of the oxidative phosphorylation (OXPHOS) machinery for oocyte health, we exposed *Xenopus* oocytes to inhibitors specific for each OXPHOS complex. We found that both early and late-stage oocytes died after treatment with inhibitors of complexes II, III, IV and V (malonate, antimycin A, KCN and *N*,*N*′-dicyclohexylcarbodiimide (DCCD), respectively). Although late-stage oocytes died after treatment with the complex I inhibitor rotenone, 78% of early oocytes survived exposure to rotenone (Fig. 2d and Extended Data Fig. 2g). The insensitivity of early oocytes to complex I inhibition indicates that they do not utilize complex I as an essential entry port for electrons.

## Mitochondrial proteome in oocytes

Mitochondria in early oocytes have an apparent lack of ROS, low membrane potential, low basal respiration rates and rotenone resistance in culture. We next investigated the mechanistic basis of this unusual mitochondrial physiology.

**Fig. 3:**
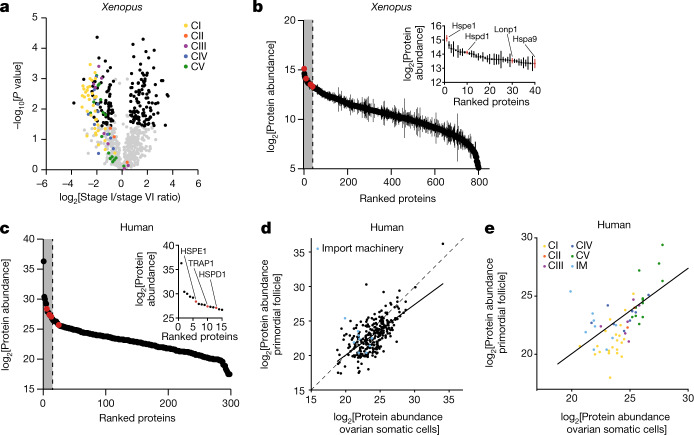
The mitochondrial proteomes of *Xenopus* and human oocytes. **a**, A volcano plot showing *P* values versus fold changes of mitochondrial proteins between early (stage I) and late (stage VI) oocytes. The subunits of the mitochondrial OXPHOS machinery are indicated in colour, according to the key in the plot. Other mitochondrial proteins significantly changing (*q* value < 0.05, >1.5-fold change) are depicted in black. *n* = 3 outbred animals, *P* values were calculated using two-sided Student’s *t*-test, and *q* values were obtained by multiple-comparison adjustment. **b**, The early *Xenopus* oocyte proteome ranked by protein abundance. The inset shows data for the top 5% most abundant proteins, corresponding to the grey area of the graph. UPR^mt^ proteins are indicated in red. The data are the mean ± s.e.m. from *n* = 3 outbred animals. **c**, The human primordial follicle proteome ranked by protein abundance. The inset shows data for the top 5% most abundant proteins, corresponding to the grey area of the graph. Oocytes were collected from ovaries of two patients and pooled together. UPR^mt^ proteins are indicated in red. **d**,**e**, Scatter plots comparing mitochondrial (**d**) and OXPHOS (**e**) protein abundance in human primordial follicles and ovarian somatic cells. The dashed line represents the identity line *x* = *y* and the solid line shows the linear regression estimate relating protein abundance between mitochondrial proteomes of primordial follicles and ovarian somatic cells. IM, import machinery.

**Extended Data Fig. 3:**
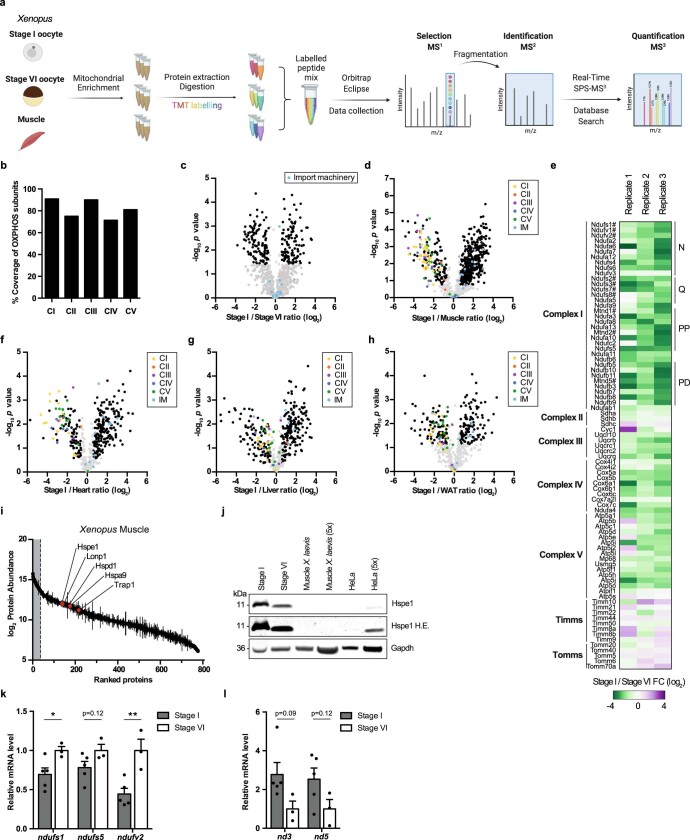
OXPHOS machinery is reduced in *Xenopus* early oocytes. **a**, A schematic representation of isobaric-tag-based quantification of the mitochondrial proteomes of early (stage I), late (stage VI) oocytes, and muscle. The image was created with BioRender.com. **b**, Percentage of proteins identified in the isobaric-tag-based quantification compared to all known subunits of OXPHOS machinery belonging to complexes I to V. **c**, A volcano plot showing *P* values versus fold changes of mitochondrial proteins between early (stage I) and late (stage VI) oocytes. Proteins significantly changing (*q* value <0.05, >1.5-fold change) are depicted in black. Subunits of mitochondrial import machinery (TIMs and TOMs) are highlighted in light blue. n=3 biological replicates. **d**, A volcano plot showing *P* values versus fold changes of mitochondrial proteins between early (stage I) oocytes and muscle. Subunits of OXPHOS and mitochondrial import machinery are highlighted in indicated colours. Other proteins significantly changing (*q* value <0.05, >1.5-fold change) are depicted in black. n=3 biological replicates. **e**, Heatmap of fold changes (early- vs late-stage oocytes) of all quantified subunits of the OXPHOS and mitochondrial import machinery. # marks core subunits of complex I. **f**, **g**, **h**, Volcano plots displaying *P* values versus fold changes of mitochondrial proteins between early (stage I) oocytes and heart (**f**), liver (**g**) and white adipose tissue (WAT) (**h**). Subunits of OXPHOS and mitochondrial import machinery are highlighted in indicated colours. Other proteins significantly changing (*q* value <0.05, >1.5-fold change) are depicted in black. n=2 biological replicates. **i**, Mitochondrial proteome in *Xenopus* muscle ranked by abundance. UPR^mt^ proteins are indicated in red. The data represent the mean ± s.e.m. from n=3 animals. **j**, Immunoblotting of Hspe1 in early (stage I), late (stage VI) oocytes, muscle and HeLa cells. Gapdh is used as a loading marker. H.E: High Exposure. One representative immunoblot from two independent experiments is shown. **k**, **l**, mRNA levels of early (stage I) and late (stage VI) oocytes of nuclear (**k**) and mitochondrial (**l**) encoded complex I subunits. The data represent the mean ± s.e.m. (n=5 for stage I and n=3 for stage VI). *P = 0.041, **P = 0.0081 using two-sided Student’s *t*-test with Šidák-Bonferroni-adjusted *P* values for multiple comparisons. CI - CV: Complex I - Complex V; IM: Import machinery. In (**c**,**d**,**f**,**g**,**h**) *P* values were calculated using two-sided Student’s *t*-test, an *q* values were obtained by multiple comparison adjustment. Source data

To do this, we purified mitochondria from early and late-stage *Xenopus* oocytes isolated from wild-type outbred animals, and performed proteomics using isobaric-tag-based quantification including muscle mitochondria as a somatic cell control (Extended Data Fig. 3a). Our efforts identified 80% of all known mitochondrial proteins (Extended Data Fig. 3b,c and Supplementary Table 1). Most ETC subunits showed a lower absolute abundance in early oocytes compared to that in late-stage oocytes (Fig. 3a), and to muscle (Extended Data Fig. 3d), which is expected owing to the presence of fewer cristae in mitochondria of early oocytes^13–15^ and compatible with their NADH levels^16^. In support of our findings with the ETC inhibitors (Fig. 2d and Extended Data Fig. 2g), the depletion of complex I in early oocytes was the most pronounced of all ETC complexes (Fig. 3a and Extended Data Fig. 3e). We reinforced this result by repeating proteomics with heart, liver and white adipose tissues (Extended Data Fig. 3f–h and Supplementary Table 2).

**Extended Data Fig. 4:**
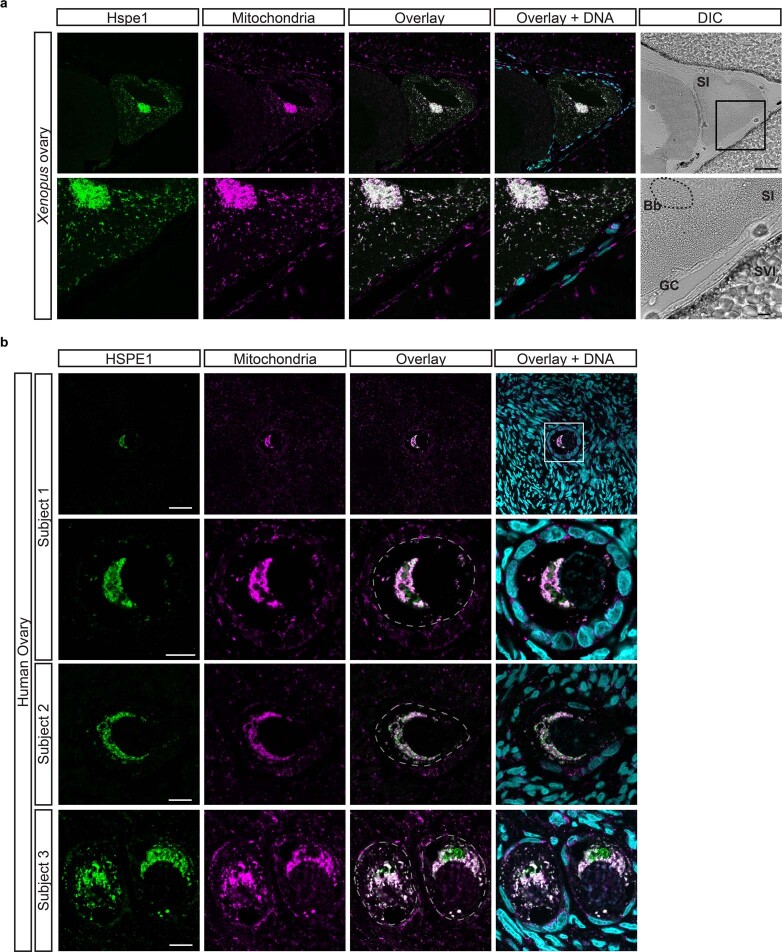
UPR^mt^ is constitutively active in early oocytes of human and *Xenopus*. **a**, **b**, Immunofluorescence of paraffin embedded sections of *Xenopus* (**a**) and human (**b**) ovaries with antibodies against HSPE1 (to monitor UPR^mt^) and ATP5A1 (to mark mitochondria). Hoechst was used to mark DNA. ATP5A1 was detected both in oocytes and somatic cells of the ovary. HSPE1, on the other hand, was so abundant in oocytes compared to surrounding cells that no signal could be detected from somatic cells without oversaturating the HSPE1 signal from early oocytes. Representative images from three independent experiments are shown. SI: early (stage I) oocyte; SVI: late (stage VI) oocyte; GC: Granulosa cells; Bb: Balbiani body. DIC, differential interference contrast. Scale bars: 50 µm (upper panel) and 10 µm (lower panel) for *Xenopus*, and 30 µm (upper panel) and 10 µm (three lower panels) for human.

Furthermore, among the most abundant proteins in the mitochondria of early oocytes were mitochondrial proteases and chaperones (Fig. 3b and Extended Data Figs. 3i,j and 4a). These proteins are upregulated after the activation of the mitochondrial unfolded protein response (UPR^mt^)^17–19^, which is often triggered by an imbalance of ETC subunits in mitochondria. Consistent with an active UPR^mt^ (ref. ^20^), nuclear transcripts encoding complex I subunits were downregulated in early oocytes whereas mitochondrially encoded transcripts of complex I did not show significant changes compared to those of late-stage oocytes (Extended Data Fig. 3k,l).

**Extended Data Fig. 5:**
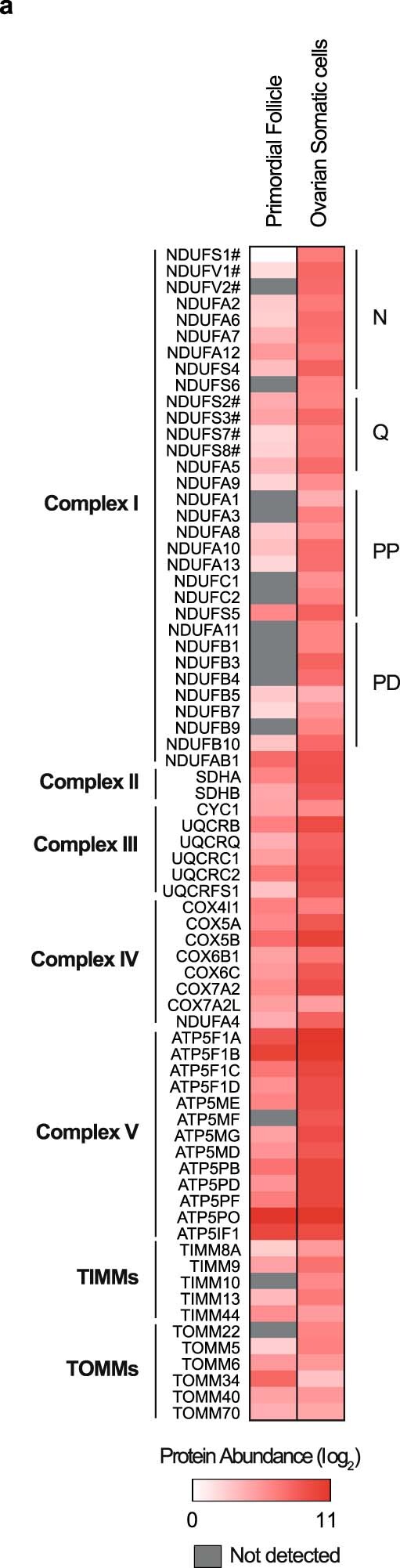
OXPHOS machinery is reduced in human early oocytes. **a**, Heatmap of normalized mitochondrial protein abundances in human primordial follicles and ovarian somatic cells organized by subunits of OXPHOS and mitochondrial import machinery. # marks core subunits of complex I. Proteins that were not identified are indicated in grey.

We next examined whether complex I subunits were also depleted in human oocytes. Early oocytes and ovarian somatic cells were isolated from ovarian cortices of patients, and analysed by label-free proteomics. We identified 40% of all known mitochondrial proteins (Supplementary Table 3). The upregulation of proteins related to UPR^mt^ was conserved in human early oocytes, and further confirmed with immunofluorescence (Fig. 3c and Extended Data Fig. 4b). An analysis of the OXPHOS machinery comparing oocytes and ovarian somatic cells revealed that, in line with the *Xenopus* data, many complex I subunits were either at very low levels or not identified in human oocytes (Fig. 3d,e and Extended Data Fig. 5a).

In conclusion, our proteomic characterization of mitochondria revealed an overall reduction of ETC subunits in early oocytes of human and *Xenopus*, with complex I levels exhibiting the strongest disproportionate depletion.

## Absence of complex I in early oocytes

Taken together, the results of our proteomics and survival experiments suggest that both early human and *Xenopus* oocytes remodel their ETC to decrease complex I levels to an extent that complex I becomes unnecessary for survival. This result is unexpected, because no other animal cell type with functioning mitochondria has been shown to be able to dispense with complex I in physiological conditions, and only one other multicellular eukaryote, the parasitic plant mistletoe, is known to dispense with complex I entirely^21^. Therefore, we directly assayed complex I assembly status and function in early oocytes, using colorimetric, spectrophotometric and metabolic assays.

**Fig. 4:**
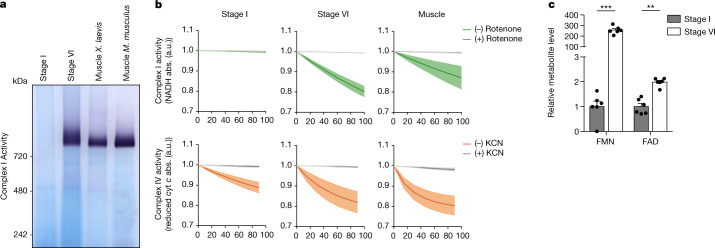
Complex I is not assembled in early oocytes. **a**, Mitochondrial fractions solubilized in *n*-dodecyl-β-d-maltoside (DDM) were resolved by BN-PAGE and complex I activity was assayed by reduction of nitro blue tetrazolium chloride (NBT) in the presence of NADH. *n* ≥ 3 (see Extended Data Fig. 6b for quantifications). **b**, Spectrophotometric analysis of complex I (green, rotenone-specific activity) and complex IV (orange, KCN-specific activity) activities in mitochondrial extracts from early (stage I) and late (stage VI) oocytes and muscle. cyt *c*, cytochrome *c*; abs, absorbance; a.u., arbitrary units. The data represent the mean ± s.e.m.; *n* = 3 biological replicates. **c**, Flavin mononucleotide (FMN) and flavin adenine dinucleotide (FAD) levels in early (stage I) and late (stage VI) *Xenopus* oocytes. The data represent the mean ± s.e.m.; *n* = 6. ***P = 6.92 × 10^−9^ and **P = 3.57 × 10^−5^ using two-sided Student’s *t*-test with Šidák–Bonferroni-adjusted *P* values for multiple comparisons. Source data

**Extended Data Fig. 6:**
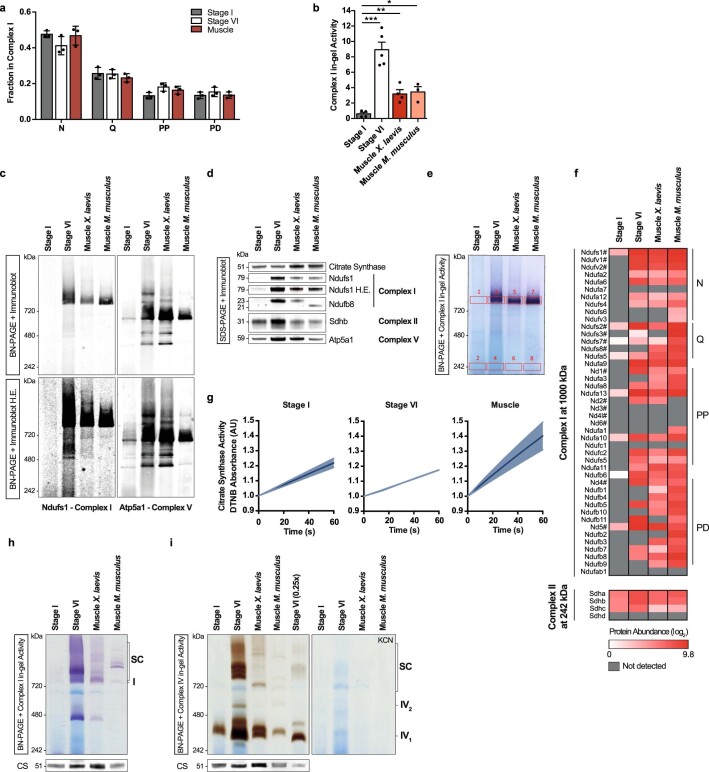
Complex I is not assembled in early oocytes. **a**, Fraction of the mean abundance of the indicated modules among all complex I modules in *Xenopus* oocytes and muscle tissue. Note that this analysis inevitably misses undetected subunits. The data represent the mean ± s.e.m. for n=3 animals. **b**, Quantification of reduced NBT intensity in BN-PAGE gels after complex I in-gel activity assays. The data represent the mean ± s.e.m. (n=5 for stage VI, n=4 for stage I and frog muscle, and n=3 for mouse muscle; biological replicates). *P = 0.0062, **P = 0.00046, and ***P = 1.44 × 10^−4^ using two-sided Student’s *t*-test with Šidák-Bonferroni-adjusted *P* values for multiple comparisons. **c**, DDM solubilized mitochondrial fractions were resolved by BN-PAGE followed by immunoblotting using antibodies against Ndufs1 and Atp5a1 to detect complexes I and V, respectively. Lower panels show high exposure (H.E.) blots. One representative immunoblot from three independent experiments is shown. **d**, SDS-PAGE and immunoblotting of mitochondrial fractions in (Fig. 4a) for complex I (Ndufs1 and Ndufb8), complex II (Sdhb) and complex V (Atp5a1) subunits, and citrate synthase as mitochondrial loading control. H.E. denotes high exposure. One representative immunoblot from three independent experiments is shown. **e**, Representation of excision sites for mass spectrometric identification of complex I and complex II subunits. **f**, Heatmap of protein abundances detected in bands excised from regions corresponding to assembled complex I, and complex II. Complex I subunits are organized by module, and # marks core subunits. Proteins that were not quantified are indicated in grey. **g**, Spectrophotometric analysis of citrate synthase activity in mitochondrial extracts from early (stage I), late (stage VI) oocytes and muscle. The data represent the mean ± s.e.m., n=3 biological replicates. **h**, **i** Digitonin solubilized mitochondrial fractions were resolved by BN-PAGE. Complex I (**h**) and complex IV (**i**) activities were assayed to detect supercomplexes (SC). Representative gels from three independent experiments are shown. Source data

We first investigated the assembly status of complex I in oocytes, which is tightly linked to its function^22^. Complex I is an approximately 1-MDa complex composed of 14 core and 31 accessory subunits in humans, some of which are essential for its assembly and function^23^. We first examined our proteomics data for any specific downregulation of a particular complex I module in early oocytes. However, levels of subunits belonging to the four major functional modules of complex I, namely N, Q, PP and PD modules, were not significantly different between *Xenopus* early and late-stage oocytes (Extended Data Fig. 6a). The size of complex I in native protein gels has been used as a tool to reveal the assembly status of the complex^22,24,25^. Thus, we compared mitochondria isolated from early oocytes to those from late-stage oocytes, and from muscle tissue of *Xenopus* and mice as somatic cell controls, by blue native polyacrylamide gel electrophoresis (BN-PAGE) followed by complex I in-gel activity assays or by an immunoblot against a complex I core subunit, Ndufs1. Notably, complex I neither was fully assembled nor exhibited any in-gel activity in early oocytes (Fig. 4a and Extended Data Fig. 6b,c). Denaturing SDS–PAGE gels also verified comparable mitochondrial loading and very low protein levels of complex I subunits in early oocytes (Extended Data Fig. 6d). To rule out any possibility of immunoblotting detection problems, areas corresponding to assembled complex I and complex II from BN-PAGE gels were analysed by proteomics (Extended Data Fig. 6e). Although complex II subunits were detected at comparable levels in all samples, most complex I subunits were not detected in early oocytes (Extended Data Fig. 6f and Supplementary Table 4). Thus, we conclude that complex I is not fully assembled in early oocytes.

In-gel activity assays detect the presence of flavin mononucleotide (FMN)-containing (sub)assemblies of complex I, but do not detect the physiological activity of the assembled complex. Therefore, we measured NADH:CoQ oxidoreductase activity in isolated mitochondrial membranes from early and late-stage oocytes, as well as muscle tissue, to measure substrate consumption by complex I, which reflects physiological activity of complex I (Fig. 4b). We also measured complex IV and citrate synthase activities to confirm the presence of mitochondrial activity in these samples. Complex IV and citrate synthase activities were detected in all three samples (Fig. 4b and Extended Data Fig. 6g). However, complex I activity was absent in early oocyte samples, in contrast to the findings for late-stage oocyte samples and muscle samples (Fig. 4b).

Finally, to validate the absence of complex I in early oocytes, we checked the levels of FMN, an integral part of complex I in early and late-stage oocytes. Although levels of another flavin nucleotide, flavin adenine dinucleotide (FAD), were within a 2-fold range between these stages, FMN levels were about 200-fold higher in late-stage oocytes, compared to the low levels detected in early oocytes (Fig. 4c). The remarkable depletion of FMN is complementary evidence supporting complex I deficiency in early oocytes.

The absence of complex I could also explain the reduced activity of other ETC complexes in early oocytes by affecting the stability of supercomplexes^26^. Assessment of supercomplex distribution showed no supercomplex formation in early oocytes, in contrast to the findings in late-stage oocytes and muscle (Extended Data Fig. 6h,i). Thus, we conclude that the absence of complex I impedes the formation of supercomplexes, which might contribute to the overall reduction of ETC activity in early oocytes.

## Complex I and ROS throughout oogenesis

We then reasoned that an absence of complex I, one of the main ROS generators in the cell, might be sufficient to explain the undetectable ROS levels in early oocytes^27^. Therefore, we studied the relationship between complex I abundance and ROS levels throughout oogenesis.

**Fig. 5:**
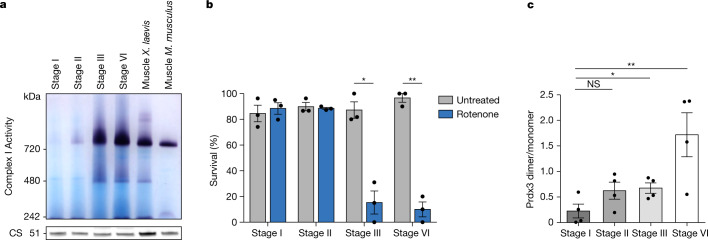
Complex I and ROS levels correlate throughout oogenesis. **a**, Mitochondrial fractions from early (stage I), maturing (stage II and stage III) and late-stage (stage VI) *Xenopus* oocytes and muscle solubilized in *n*-dodecyl-β-d-maltoside (DDM) were resolved by BN-PAGE and complex I activity was assayed. One representative gel from three independent experiments is shown. CS, citrate synthase. **b**, Overnight survival of early (stage I), maturing (stage II and III) and late-stage (stage VI) *Xenopus* oocytes after treatment with the complex I inhibitor rotenone (5 µM). At least 10 oocytes were incubated per condition. The data represent the mean ± s.e.m.; *n* = 3 biological replicates. *P = 0.0028 and **P = 0.0002 using two-sided Student’s *t*-test with Šidák–Bonferroni-adjusted *P* values for multiple comparisons. **c**, Prdx3 dimer/monomer ratio assessed in oocytes in the indicated stages of oogenesis. The data represent the mean ± s.e.m; *n* = 4 biological replicates. NS, not significant (*P* = 0.1128), *P = 0.0376 and **P = 0.0164 using two-sided Student’s *t*-test with Šidák–Bonferroni-adjusted *P* values for multiple comparisons. Source data

First, we investigated the assembly of complex I during oogenesis. Complex I activity was barely detectable in stage II oocytes, but peaked and plateaued in maturing (stage III) oocytes (Fig. 5a). We then assessed the survival of oocytes in the presence of rotenone throughout oogenesis. The overnight survival of oocytes in rotenone was consistent with their levels of assembled complex I: stage I and II oocytes survived in the presence of rotenone whereas maturing and mature oocytes died (Fig. 5b). Hence, we conclude that complex I is assembled and fully functional in maturing (stage III) and late-stage oocytes but absent in early oocytes.

**Extended Data Fig. 7:**
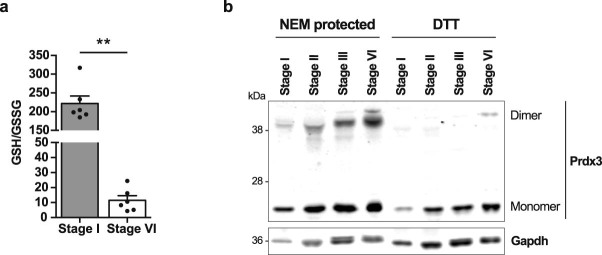
Complex I and ROS levels throughout oogenesis. **a**, GSH/GSSG ratio in early (stage I) and late-stage (stage VI) oocytes. The data represent the mean ± s.e.m., n=6. ** *P* = 1.24 × 10^−6^ using two-sided Student’s *t*-test. **b**, Immunoblotting of Prdx3 in early (stage I), maturing (stage II and III), and late-stage (stage VI) oocyte extracts processed in non-reducing (NEM protected) or reducing (DTT) conditions. Monomeric and dimeric Prdx3 are indicated. Gapdh was used as a loading control. One representative immunoblot from four independent experiments is shown. Source data

Second, we investigated whether the assembly of complex I throughout oogenesis was accompanied by the production of ROS in oocytes. The opacity of maturing *Xenopus* oocytes impedes the use of most fluorescent ROS markers. Therefore, we turned to known metabolic and protein 'sentinels' of ROS levels in cells and evaluated the redox state of glutathione^28–30^ and mitochondrial peroxiredoxin 3 (Prdx3) in oocytes. We found that ratio of reduced glutathione to oxidized glutathione was 20-fold higher in early oocytes compared to that in late-stage oocytes (Extended Data Fig. 7a), indicating a reduced cellular redox state in early oocytes, consistent with oocytes having undetectable levels of ROS. Next, we checked the redox state of Prdx3 during oogenesis. Peroxiredoxins dimerize in the presence of peroxide, and thus, the ratio of peroxiredoxin dimers to monomers correlates with the level of cellular peroxide^31–33^. Prdx3 dimerization increased throughout oogenesis, from negligible levels in early (stage I) oocytes to the highest measured level in late-stage (stage VI) oocytes (Fig. 5c and Extended Data Fig. 7b). Stage II oocytes, in which complex I activity is very low (Fig. 5a), showed a nonsignificant increase in dimer/monomer ratio (Fig. 5c).

The timing of complex I assembly and the increase in ROS levels correlate: ROS start to build up as soon as complex I is assembled in oocytes. On the basis of these results, we speculate that the maturation of oocytes involves a slow, gradual transition to a metabolism that involves a functional complex I.

Combining the in vivo evidence with proteomics and biochemical assays in vitro, our results demonstrate that early oocytes avoid ROS by eliminating one of the main ROS generators in the cell, mitochondrial complex I. Complex I subunits are reduced to such low levels that complex I cannot be fully assembled, nor can its activity be detected in early oocytes. This reveals a new strategy used by *Xenopus* and most likely human oocytes to maintain a low-ROS-producing mitochondrial metabolism. Although quiescence is associated with ETC remodelling in *Drosophila* oocytes^34^, to our knowledge, vertebrate early oocytes are the first and only physiological cell type in animals that exist without a functional mitochondrial complex I.

## Discussion

Here we have shown that dormancy involves survival with an inactive mitochondrial complex I. By shutting down complex I and keeping the rest of the OXPHOS system active, early oocytes keep their mitochondria polarized to support the synthesis of haeme, essential amino acids and nucleotides, while keeping their activity low to avoid ROS. Other quiescent cells, such as neuronal and haematopoietic stem cells, exhibit similarly low ROS levels, and reduced ETC activity^9,35^, raising the possibility that this regulatory mechanism might be utilized by other cell types. Furthermore, UPR^mt^ is activated in early oocytes (Fig. 3b,c and Extended Data Fig. 4), probably in response to an imbalance of ETC complexes caused by the absence of complex I. Given that UPR^mt^ activation itself is sufficient to increase the lifespan of *Caenorhabditis elegans* and mouse^17–19^, we speculate that complex I inhibition further enhances the longevity of oocytes through its downstream activation of UPR^mt^. The causal relationships between these interacting factors and oocyte lifespan remain a fascinating future direction to investigate.

Severe sample limitations prevent biochemical assays of human oocytes—30 thousand donor ovaries would be required for one experiment to directly measure complex I function using current technologies. Ideally, future methodological developments will allow direct evaluation of complex I activity in human oocytes. It would also be interesting to investigate whether similar mechanisms apply in the oocytes of other mammals such as mice. Until then, we rely on proteomics, imaging and the activation of downstream pathways (UPR^mt^) that suggest that complex I is also absent in human primordial oocytes. Moreover, the absence of complex I in early oocytes can also explain why complex-I-related mitochondrial pathologies (such as Leber's hereditary optic neuropathy) do not lead to subfertility or selection against homoplasmic mitochondrial DNA mutations that occur in other types of ETC dysfunction^36–38^. As the oogenic mitochondrial bottleneck occurs in early oogenesis^39^, there would not be a selective pressure against mutations affecting an inactive complex I.

Our findings reveal yet another unique aspect of physiology that oocytes have evolved to balance their essential function of beginning life with the requirement for longevity. This raises the question whether complex I deficiency in primordial oocytes can be exploited for other purposes. Some cancers seen in young women are highly treatable; however, their treatment leads to a severe reduction of the ovarian reserve and reduced prospects of motherhood. Drugs against complex I exist, and are already proposed for cancer treatments^40^. Future studies will show whether repurposing complex I antagonists can improve chemotherapy-related infertility, and thus life quality of young female cancer survivors.

## Methods

### Ethics

Ethical committee permission to work with primordial oocytes from human ovary samples was obtained from the Comité Étic d’Investigació Clínica CEIC-Parc de Salut MAR (Barcelona) and Comité Ético de Investigación Clínica–Hospital Clínic de Barcelona with approval number HCB/2018/0497. Written informed consent was obtained from all participants before their inclusion in the study.

Animals used in this study were housed in the Barcelona Biomedical Research Park, accredited by the International Association for Assessment and Accreditation of Laboratory Animal Care. Animal euthanasia was performed by personnel certified by the competent authority (Generalitat de Catalunya) and conformed to the guidelines from the European Community Directive 2010/63 EU, transposed into Spanish legislation on RD 53/2013 for the experimental use of animals.

### Animal models

*Xenopus laevis* adult females of between 2 and 4 years old were purchased from Nasco and maintained in water tanks in the following controlled conditions: 18–21 °C, pH 6.8–7.5, O_2_ 4–20 ppm, conductivity 500–1,500 µs, ammonia <0.1 ppm. The C57BL/6J mice used in the experiments were purchased from Charles River Laboratories and maintained in the Animal Facility of the Barcelona Biomedical Research Park under specific-pathogen-free conditions at 22 °C with 40–60% humidity, in a 12 h light/dark cycle, and with access to food and water ad libitum. Female mice of 7 weeks of age were used for extracting muscle tissue.

### Oocyte isolation and culture

#### Human primordial oocytes

Ovaries were provided by the gynaecology service of Hospital Clinic de Barcelona, from women aged 19 to 34 undergoing ovarian surgery and were processed as previously described^6^. Briefly, ovarian cortex samples were digested in DMEM containing 25 mM HEPES and 2 mg ml^−1^ collagenase type III (Worthington Biochemical, LS004183) for 2 h at 37 °C with occasional swirling. Individual cells were separated from tissue fragments by sedimentation, and collagenase was neutralized by adding 10% FBS (Thermo, 10270106). Follicles were picked manually under a dissecting microscope. All human oocyte imaging experiments were conducted in DMEM/F12 medium (Thermo, 11330-032) containing 15 mM HEPES and 10% FBS (Thermo, 10270106).

#### *Xenopus* oocytes

Ovaries were dissected from young adult (aged 3 to 5 years) female *X. laevis* that had undergone euthanasia by submersion in 15% benzocaine for 15 min. Ovaries were digested using 3 mg ml^−1^ collagenase IA (Sigma, C9891-1G) in Marc's modified Ringer's (MMR) buffer by gentle rocking until dissociated oocytes were visible, for 30 to 45 min. The resulting mix was passed through two sets of filter meshes (Spectra/Mesh, 146424 and 146426). All washes were performed in MMR. For live-imaging experiments with intact granulosa cells, oocytes were transferred to oocyte culture medium (OCM)^41^ at this stage. For the rest of the experiments, oocytes were stripped of accompanying granulosa cells by treatment with 10 mg ml^−1^ trypsin in PBS for 1 min, followed by washes in MMR. Removal of granulosa cells was confirmed by Hoechst staining of a small number of oocytes.

### HeLa cell culture

HeLa cells were obtained from ATCC (CCL2), authenticated based on morphological inspection and confirmed to be mycoplasma negative. Cells were grown in DMEM (Thermo, 41965039) supplemented with 1 mM sodium pyruvate (Thermo, 11360070) and 10% FBS (Thermo, 102701060).

### Live-cell imaging

Human or *Xenopus* early oocytes were labelled in their respective culture medium (see above). Human oocytes were imaged using a 63× water-immersion objective (NA 1.20, Leica, 506346) with an incubation chamber maintained at 37 °C and 5% CO_2_. Frog oocytes were imaged using a 40× water-immersion objective (NA 1.10, Leica, 506357) in OCM at room temperature and atmospheric air, unless stated otherwise. All images were acquired using a Leica TCS SP8 microscope with the LAS X software (Leica, v3.5.5.19976). Mean fluorescence intensities in granulosa cells and oocytes were quantified using Fiji software.

#### ROS probes

Oocytes and associated granulosa cells were incubated in 500 nM MitoTracker Red CM-H2Xros (Thermo, M7513) for 30 min, 5 µM MitoSOX Red (Thermo, M36008) for 10 min, or 5 µM CellROX for 30 min. Cells were then washed and imaged in 35-mm glass-bottom MatTek dishes in culture medium, except for CellROX labelling, for which MMR was used for imaging to satisfy the manufacturer’s instructions.

#### Mitochondrial membrane potential probes

Oocytes and associated granulosa cells were labelled for 30 min in 500 nM tetramethylrhodamine ethyl ester perchlorate (TMRE) (Thermo, T669), or 45 min in 4 µM JC-1 (Abcam, ab141387). Cells were then washed and imaged in 35-mm glass-bottom MatTek dishes.

### Oxygen consumption rate

Oxygen consumption rate (OCR) of *Xenopus* oocytes was measured using a Seahorse XFe96 Analyser (Agilent) with Seahorse Wave software (Agilent, v2.6). Granulosa-cell-stripped oocytes were placed in XFe96 culture plates immediately after their isolation in Seahorse XF DMEM medium pH 7.4 supplemented with 10 mM glucose, 1 mM pyruvate and 2 mM glutamine (Agilent; 103015-100, 103577-100, 103578-100 and 103579-100). A cartridge was loaded with concentrated inhibitor solution to achieve 5 µM oligomycin, 2 µM carbonyl cyanide 4-(trifluoromethoxy) phenylhydrazone or a combination of 0.5 µM rotenone and 0.5 µM antimycin A. Mock medium injections were performed to account for inhibitor-independent decline in OCR. For each sequential injection, at least 4 measurement cycles were acquired consisting of 20 s mix, 90 s wait and 3 min measure, in at least 3 replicates. For basal and maximal respiration rates, assay-independent OCR decline was corrected, and non-mitochondrial respiration (resistant to rotenone–antimycin mix) was subtracted. OCR measurements for growing oocytes (stage III; with a diameter of 450–600 µm (ref. ^42^)) had to be performed statically because the probe of the analyser compressed and destroyed these large oocytes in long-term measurements. For growing (stage III) oocytes, OCR was acquired during 5 cycles per well, each cycle being 20 s mix, 90 s wait and 3 min measure, in at least 4 replicates. The well size imposed a technical limitation on the maximum number of oocytes per well (100 early and 8 growing oocytes); thus, respiration data were normalized for the total protein amount per sample.

### Treatments with OXPHOS inhibitors

At least 50 stage I and stage II, 20 stage III and 10 stage VI oocytes were assayed per condition. Oocytes were placed in 35-mm glass-bottom dishes (MatTek) and incubated for 16 h at 18 °C in OCM with or without the addition of the indicated mitochondrial inhibitors at the following concentrations: 5 µM rotenone (Sigma, R8875), 50 mM malonic acid (Sigma, M1296), 5 µM antimycin A (Abcam, ab141904), 50 mM potassium cyanide (KCN; Merck Millipore, 1049670100), 200 µM *N*,*N*′-dicyclohexylcarbodiimide (DCCD) (Sigma, D80002) and 30 µM carbonyl cyanide *m*-chlorophenyl hydrazone (CCCP) (Abcam, ab141229). Survival was assessed by counting the number of oocytes with intact morphology before and after treatments. Cell death in stage III to VI oocytes was recognized by the development of a mottling pattern in the pigmentation^43^. Images were taken by a Leica IC90 E stereoscope.

Early (stage I) oocytes were treated with 10 µM menadione (Sigma, M5625) or left untreated, for 2 h in OCM, and washed into fresh OCM. Untreated oocytes were labelled with wheat germ agglutinin 488 (Biotium, 29022-1) to mark their plasma membrane and mixed with menadione-treated oocytes in a glass-bottom MatTek dish 4 h after menadione was removed. The mixed population of oocytes were then labelled with MitoSOX and imaged. At least 50 stage I and II oocytes and at least 10 stage III and VI oocytes were treated with 10 µM menadione (Sigma, M5625) in the presence or in the absence of 10 mM *N*-acetyl cysteine (NAC) (Sigma, A9165). After 2 h, menadione was removed and *N*-acetyl cysteine was retained for an overnight incubation. Survival was determined by counting the number of oocytes immediately before menadione treatment (*t* = 0) and after 16 h in recovery.

### Mitochondrial-enriched extracts

Mitochondrial-enriched fractions were obtained as described previously for gastrocnemius muscle and with minor adaptations for oocyte samples^44^. Freshly isolated early oocytes from *Xenopus* were lysed in mitochondria buffer (250 mM sucrose, 3 mM EGTA, 10 mM Tris pH 7.4), and spun at low speed to remove debris. The resulting supernatant was centrifuged at 20,000*g* for 20 min at 4 °C. Late-stage oocytes were spin-crashed, and yolk-free fraction was combined 1:1 with mitochondria buffer and centrifuged at 20,000*g* for 20 min at 4 °C to pellet mitochondria. Mitochondrial pellets from early and late-stage oocytes were resuspended in mitochondria buffer and subjected to DNase treatment for 10 min and proteinase K treatment for 20 min. Phenylmethylsulfonyl fluoride was added to stop proteolytic activity and samples were centrifuged again at 20,000*g* for 20 min at 4 °C. Protein concentration was estimated and aliquots of crude mitochondria were stored at −80 °C until use.

### Spectrometric assessment of enzymatic activities of mitochondrial complexes

The specific activities of mitochondrial complex I, complex IV and citrate synthase were determined as described before with minor modifications^45^. Briefly, mitochondrial extracts were subjected to three freeze–thaw cycles in hypotonic buffer (10 mM Tris-HCl) before activity analysis using an Infinite M200 plate reader (Tecan) with Tecan i-control software (Tecan, v3.23) in black-bottom 96-well plates (Nunc) at 37 °C. For complex I NADH:CoQ activity assessment, reaction solutions (50 mM KP pH 7.5, 3 mg ml^−1^ BSA, 300 µM KCN and 200 µM NADH) with or without rotenone (10 µM) were distributed into each well first. Mitochondrial extracts were then added and NADH absorbance at 340 nm was measured for 2 min to establish baseline activity. The reaction was then started by the addition of ubiquinone (60 µM). NADH absorbance was recorded for 15 min every 15 s.

For complex IV activity assessment, reaction solutions (50 mM KP pH 7, 60 µM reduced cytochrome *c*) with or without KCN (600 µM) were distributed into each well first, and absorbance of reduced cytochrome *c* at 550 nm was recorded for 2 min to establish baseline oxidation. Mitochondrial extracts were then added and absorbance was measured for 15 min every 15 s.

For citrate synthase activity, reaction solution (100 µM Tris pH 8, 0.1% Triton X-100, 100 µM DTNB and 300 µM acetyl CoA) was distributed into each well first. Mitochondrial extracts were then added and absorbance at 410 nm was measured for 2 min to set the baseline; then the reaction was started by addition of the substrate oxaloacetic acid (500 µM). Production of TNB (yellow) was recorded by measuring the absorbance at 410 nm for 15 min every 15 s. Enzymatic assays were plotted with the baseline represented as 1 for simplicity.

### Denaturing SDS gel electrophoresis

Oocytes were collected after isolation, frozen in liquid nitrogen and kept at −80 °C until further use. Samples were processed as described previously^46^. Gastrocnemius total homogenates were obtained as described previously^47^. HeLa cells were lysed in RIPA buffer (50 mM Tris-HCl, 150 mM NaCl, 1% Nonidet P-40, 01% SDS and 1 mM EDTA, supplemented with protease inhibitor cocktail (Complete Roche Mini, 1 tablet per 50 ml)) and spun at 20,000*g* to eliminate cell debris. Oocyte lysates for determination of the redox state of peroxiredoxin were protected against artefactual oxidation by alkylation as described previously^48^, but in OCM. Cell lysates or mitochondrial-enriched fractions were resolved by SDS–PAGE using 4–12% NuPAGE Bis-Tris gels.

### BN-PAGE electrophoresis, and in-gel activity assays

Mitochondrial content in samples of different cell types (different stages of oocytes and muscle tissue) was first assessed by western blotting for their citrate synthase levels (Supplementary Figs. 1b and 2c,d). Next, similar amounts of mitochondrial fractions were solubilized in 1% *n*-dodecyl-β-d-maltoside (DDM) or digitonin, and were resolved in the native state using NativePAGE 3–12% Bis-Tris (Thermo, BN1001BOX) gradient gels as described before^49^. The left part of the gel was cut and stained with Coomassie (InstantBlue, Sigma) after BN-PAGE to reveal the native protein molecular weight marker protein (Supplementary Figs. 1a,b and 2a,c,d). Complex I and complex IV activity in-gel assays were performed as described previously^24^. Briefly, immediately after the run, BN-PAGE gels were incubated in assay solution: for complex I in 2 mM Tris pH 7.4, 0.1 mg ml^−1^ NADH and 2.5 mg ml^−1^ nitro blue tetrazolium chloride (NBT) to asses NADH:FMN electron transfer, denoted by the appearance of dark purple colour; and for complex IV in 10 mM phosphate buffer pH 7.4, 1 mg ml^−1^ cytochrome *c* and 0.5 mg ml^−1^ of 3,3′-diaminobenzidine (DAB) in the presence or absence of 0.6 mM KCN to assess the specific cytochrome *c* oxidation, denoted by the appearance of dark brown colour. The intensities of reduced nitro blue tetrazolium chloride (NBT) were normalized to citrate synthase levels of the same samples, detected by SDS–PAGE followed by immunoblotting. Gels were imaged using an Amersham Imager (GE Healthcare; Supplementary Figs. 1 and 2). Intensity measurements were performed using Fiji software.

### Immunoblot analysis

Denaturing SDS–PAGE gels were transferred to nitrocellulose membranes through wet transfer using a Mini Trans-Blot Cell (Bio-Rad). Membranes were blocked in Intercept (TBS) Blocking Buffer (LI-COR), and incubated overnight at 4 °C with primary antibodies diluted in Intercept 0.05% Tween-20 as follows: anti-ATP5A1 (Abcam; ab14748; 1:1,000), anti-citrate synthase (Abcam, ab96600; 1:1,000), anti-GAPDH (Thermo, AM4300; 1:5,000), anti-HSPE1 (Thermo, PA5-30428; 1:1,000), anti-NDUFB8 (Abcam, ab110242; 1:1,000), anti-NDUFS1 (Abcam, ab169540; 1:1,000), anti-PRDX3 (Abcam, ab73349; 1:1,000) and anti-SDHB (Abcam, ab14714; 1:1,000). Primary antibodies were washed with TBS-T (0.05% Tween-20) and membranes were incubated in the secondary antibodies anti-mouse IgG DyLight 680 (Thermo, 35518; 1:10,000) or anti-rabbit IgG DyLight 800 4× PEG (Thermo, SA5-35571; 1:10,000). After washing, membranes were imaged by a near-infrared imaging system (Odyssey LI-COR) with Image Studio software (Li-COR, v5.2; Supplementary Figs. 1 and 2). Densitometric analysis of immunoblotting images was performed using Fiji software.

BN-PAGE gels were transferred to polyvinylidene fluoride (PVDF) membranes using a Mini Trans-Blot Cell (Bio-Rad). After wet transfer, polyvinylidene fluoride (PVDF) membranes were destained in methanol, blocked and incubated with antibodies against NDUFS1 (Abcam, ab169540; 1:1,000) and ATP5A1 (Abcam, ab14748; 1:1,000) for complex I and complex V immunodetection, respectively (Supplementary Fig. 2a).

### Transcript levels

RNA from early oocytes and spin-crashed yolk-free late-stage oocyte lysates was extracted using TRI reagent (Sigma, T9424) followed by RNeasy and Oligotex mRNA column (Qiagen) purification, following the manufacturer’s instructions. cDNA was synthesized with a First Strand cDNA synthesis kit (Thermo, K1612). Quantitative real-time PCR was performed using SYBR Green I Master (Roche, 04887352001) in a LightCycler 480 with LightCycler software v1.5.1 (Roche); with the following pairs of primers: *ndufs1* forward: 5′-GGTGCGGTATGATGATGTGG-3′, reverse: 5′-ACAGCTTTCACACACTTGGC-3′; *ndufs5* forward: 5′-GTCCGAAAGTTGTGCAGTCA-3′, reverse: 5′-CGGATCTGCCCAATTCCATG-3′; *ndufv2* forward: 5′-GCATACAATGGAGCAGGTGG-3′, reverse: 5′-CATCCATGCTGTCTCTGTGC-3′; *mt-nd3* forward: 5′-ATTTGATCCTCTGGGCTCTG-3′, reverse: 5′-AGCGCAATCTCTAGGTCAAA-3′; *mt-nd5* forward: 5′-GGTCATCCACGATCAAATCCA-3′, reverse: 5′-ACCGAAACGATAATAGCTGCC-3′; *gapdh* forward: 5′-AGTTATCCCTGAGCTGAACG-3′, reverse: 5′-CTGATGCAGTCTTAATGGCG-3′; *mt-rnr2* forward: 5′-ACTACCCGAAACTAAGCGAG-3′, reverse: 5′-ATCTTCCCACTCTTTTGCCA-3′. Nuclear-DNA-encoded genes were normalized to *gapdh* levels and mitochondrial-DNA-encoded genes were normalized to *mt-rnr2*.

### Measurement of FMN and glutathione

Samples were prepared using the automated MicroLab STAR system from Hamilton Company in the presence of recovery standard for quality control by Metabolon. After protein precipitation in methanol, metabolites were extracted and analysed by ultrahigh-performance liquid chromatography with tandem mass spectrometry by negative ionization. Raw data were extracted, peak-identified and processed for quality control using Metabolon’s hardware and software.

### Immunostaining paraffin ovary sections

Human and frog ovaries were fixed in 4% PFA in PBS overnight at 4 °C, washed, embedded in paraffin blocks and cut into 5 µM sections. After deparaffinization, antigen retrieval was performed by heating the slides for 15 min in 10 mM sodium citrate at pH 6. Sections were blocked and permeabilized in 3% BSA, 0.05% Tween-20 and 0.05% Triton X-100 for 1 h at room temperature. Sections were incubated overnight at 4 °C in the presence of primary antibodies (1:100): anti-ATP5A1 (Abcam, ab14748) and anti-HSPE1 (Thermo, PA5-30428); then 2 h at room temperature with secondary antibodies (1:500). Antibodies and dyes used were as follows: goat anti-rabbit Alexa488 or Alexa555 (1:500, Thermo, A-11008, A-21428), goat anti-mouse Alexa647 (Thermo, A21236) and Hoechst dye (1:500, Abcam, ab145597). A droplet of mounting medium (Agilent, S302380) was added onto the section before imaging using the LAS X software (Leica, v3.5.5.19976) in a Leica TCS SP8 microscope equipped with 40× (NA 1.30, Leica 506358) and 63× (NA 1.40, Leica 506350) objectives.

### Statistics and reproducibility

Sample sizes were chosen based on published studies to ensure reliable statistical testing and to account for variability among outbred populations. Experimental limitations were also taken into account, such as the number of primordial oocytes that could be obtained from human ovaries. All experiments were performed on isolated oocytes or tissues. Sample randomization was performed by two means. First, all outbred frogs used in this study were chosen by blinded animal facility personnel without our knowledge. Second, all isolated oocytes or tissue samples were first grouped together and then randomly distributed to  different experimental groups. Blinding during data collection was not required as standard experimental procedures were applied for different groups, such as western blots and immunohistochemistry. Blinding during data analysis was performed in oocyte survival experiments by involving multiple lab members for analysing blinded datasets. Blinding for the analysis of other experiments was not required since the different experimental groups were analysed using the same parameters. All data are expressed as mean ± s.e.m. A simple linear regression was performed to fit a model between the mitochondrial protein abundances of primordial follicle and ovarian somatic cell samples (Fig. 3d,e). Unpaired two-tailed Student’s *t*-test was used in all other analysis, *P* values are specified in figure legends, and those <0.05 were considered significant. Multiple *t*-tests were used in Figs. 1d, 4c and 5b,c and Extended Data Figs. 2c,d, 3k,l and 6b, and were corrected by the Šidák–Bonferroni method using GraphPad Prism. In *Xenopus* proteomics experiments, *q* values were calculated as adjusted *P* values and significance was considered for *q* value < 0.05 for comparing protein levels. A fold-change heatmap was generated using JMP (version 13.2) software. For Extended Data Fig. 6f, we excised the indicated bands in Extended Data Fig. 6e from one of three gels represented in Fig. 4a; gel-identification MS was performed once.

### MS

#### Sample preparation

For isobaric-tag-based quantification for *Xenopus*, mitochondrial extracts from early (stage I) oocytes, late (stage VI) oocytes, gastrocnemius muscle, heart, liver and white adipose tissues were processed in two parallel experiments: stage I, stage VI and muscle in triplicates; and stage I, heart, liver and white adipose tissue in duplicates. Samples were quantified and 100 μg of each sample was processed with slight modifications from ref. ^46^. In brief, methanol-precipitated proteins were dissolved in 6 M guanidine hydrochloride (GuaCl). Samples were then digested with LysC (20 ng µl^−1^) in 2 M GuaCl overnight at room temperature. The next morning, samples were further diluted to 0.5 M GuaCl and digested with trypsin (10 ng µl^−1^) and further LysC (20 ng µl^−1^) for 8 h at 37 °C. Later, samples were speed-vacuumed, and the resulting pellet was resuspended in 200 mM EPPS pH 8.0. Ten-microlitre volumes of tandem mass tag (TMT) stock solutions (20 µg µl^−1^ in acetonitrile) were added to 50 μl of samples, and samples were incubated 3 h at room temperature. The TMT reaction was quenched with a 0.5% final concentration of hydroxylamine. The samples were combined in one tube, acidified by 10% phosphoric acid, and subjected to a MacroSpin C18 solid-phase extraction (The Nest Group) to desalt and isolate peptides. TMT mixes were fractionated using basic pH reversed-phase fractionation in an Agilent 1200 system. Fractions were desalted with a MicroSpin C18 column (The Nest Group) and dried by vacuum centrifugation^50^.

For label-free proteomics for human oocytes, human primordial follicles and ovarian somatic cells were collected from two individuals who underwent ovarian surgery. Samples were dissolved in 6 M GuaCl pH 8.5, diluted to 2 M GuaCl and digested with LysC (10 ng µl^−1^) overnight. Samples were further diluted down to 0.5 M GuaCl and digested with LysC (10 ng µl^−1^) and trypsin (5 ng µl^−1^) for 8 h at 37 °C. Samples were acidified by 5% formic acid and desalted with home-made C18 columns.

For detection of complex I and II subunits from BN-PAGE gels, gel bands were destained, reduced with dithiothreitol, alkylated with iodoacetamide and dehydrated with acetonitrile for trypsin digestion. After digestion, peptide mix was acidified with formic acid before analysis through liquid chromatography with MS/MS.

#### Chromatographic and MS analysis

TMT and label-free samples were analysed using a Orbitrap Eclipse mass spectrometer (Thermo) coupled to an EASY-nLC 1200 (Thermo). Peptides were separated on a 50-cm C18 column (Thermo) with a gradient from 4% to 32% acetonitrile in 90 min. Data acquisition for TMT samples was performed using a Real Time Search MS3 method^51^. The scan sequence began with an MS1 spectrum in the Orbitrap. In each cycle of data-dependent acquisition analysis, following each survey scan, the most intense ions were selected for fragmentation. Fragment ion spectra were produced through collision-induced dissociation at a normalized collision energy of 35% and they were acquired in the ion trap mass analyser. MS2 spectra were searched in real time with data acquisition using the PHROG database^52^ with added mitochondrially encoded proteins. Identified MS2 spectra triggered the submission of MS3 spectra that were collected using the multinotch MS3-based TMT method^53^.

Label-free samples were acquired in data-dependent acquisition mode and full MS scans were acquired in the Orbitrap. In each cycle of data-dependent acquisition analysis, the most intense ions were selected for fragmentation. Fragment ion spectra were produced through high-energy collision dissociation at a normalized collision energy of 28%, and they were acquired in the ion trap mass analyser.

Gel bands were analysed using a LTQ-Orbitrap Velos Pro mass spectrometer (Thermo) coupled to an EASY-nLC 1000 (Thermo). Peptides were separated on a 25-cm C18 column (Nikkyo Technos) with a gradient from 7% to 35% acetonitrile in 60 min. The acquisition was performed in data-dependent acquisition mode and full MS scans were acquired in the Orbitrap. In each cycle, the top 20 most intense ions were selected for fragmentation. Fragment ion spectra were produced through collision-induced dissociation at a normalized collision energy of 35%, and they were acquired in the ion trap mass analyser.

Digested bovine serum albumin was analysed between each sample and QCloud (ref. ^54^) was used to control instrument performance.

#### Data analysis

Acquired spectra were analysed using the Proteome Discoverer software suite (v2.3, Thermo) and the Mascot search engine (v2.6, Matrix Science^55^). Label-free data were searched against the SwissProt Human database. Data from the gel bands were searched against a custom PHROG database^52^ that includes 13 further entries that correspond to mitochondrially encoded proteins for the *Xenopus* samples and the SwissProt mouse database for the mouse samples. TMT data were searched against the same custom 'PHROG' database. False discovery rate in peptide identification was set to a maximum of 5%. Peptide quantification data for the gel bands and the label-free experiments were retrieved from the 'Precursor ion area detector' node. The obtained values were used to calculate an estimation of protein amount with the top3 area, which is the average peak area of the three most abundant peptides for a given protein. For the TMT data, peptides were quantified using the reporter ion intensities in MS3. Reporter ion intensities were adjusted to correct for the isotopic impurities of the different TMT reagents according to the manufacturer's specifications. For final analysis, values were transferred to Excel. For all experiments, identified proteins were selected as mitochondrial if they were found in MitoCarta 3.0 (ref. ^56^). MS3 spectra with abundance less than 100 or proteins with fewer than 2 unique peptides were excluded from the analysis. Each TMT channel was normalized to total mitochondrial protein abundance. A total of 926 mitochondrial proteins were identified (and 807 quantified) in 3 biological replicates from wild-type outbred animals, representing 80% of known mitochondrial proteins (Supplementary Table 1 and Extended Data Fig. 3b). Although the mitochondrial proteome in diverse cell types could be quite different^57^, we found comparable levels of mitochondrial housekeeping proteins (such as the import complexes TIMMs and TOMMs) across different maturity stages (Extended Data Fig. 3c and Supplementary Table 1), enabling us to compare and contrast changes in other pathways.

For human somatic cell samples, we analysed three dilutions: the 1× reference had a similar level of protein loading to that of the primordial follicle sample (0.55 µg total protein); a twofold dilution (0.25 µg total protein); and a fivefold dilution (0.1 µg total protein). In scatter plots (Fig. 3d,e), we estimated differences in mitochondrial complex I protein abundance using the twofold somatic cell dilution, a conservative approach that compared primordial follicle samples (0.55 µg total protein) to somatic cells half their loading concentration (0.25 µg total protein), nevertheless observing similar levels of the mitochondrial import machinery subunits TOMMs and TIMMs. The fivefold-dilution somatic cell sample was useful for establishing detection limits; indeed, many complex I subunits absent in oocytes were detected with high confidence even at this dilution. In the heatmap (Extended Data Fig. 5), we considered normalizing our data using the mitochondrial loading controls citrate synthase and COX4I1 to estimate differences in protein abundance. The abundance of COX4I1 fell within the linear range of our proteomic methodology (*R*^2^ = 0.99), in contrast to that for citrate synthase (*R*^2^ = 0.89) whose higher abundance led to measurement saturation at higher concentrations. Therefore, COX4I1 was chosen to normalize protein abundances in the heatmap representation. We identified 454 mitochondrial proteins (Supplementary Table 3; 298 and 397 proteins were quantified for early oocyte and somatic cell samples, respectively), representing 40% of all known mitochondrial proteins. Here too, levels of the mitochondrial import proteins TIMMs and TOMMs were similar between oocytes and ovarian somatic cells (Fig. 3d,e), demonstrating an equivalent mitochondrial abundance that facilitated comparison of protein levels between different cell types.

### Reporting summary

Further information on research design is available in the Nature Research Reporting Summary linked to this paper.

## Online content

Any methods, additional references, Nature Research reporting summaries, source data, extended data, supplementary information, acknowledgements, peer review information; details of author contributions and competing interests; and statements of data and code availability are available at 10.1038/s41586-022-04979-5.

## Supplementary information

Supplementary Information This file contains Supplementary Figs. 1 and 2 and legends for Supplementary Tables 1–4.

Reporting Summary

Supplementary Table 1

Supplementary Table 2

Supplementary Table 3

Supplementary Table 4

## Data Availability

Isobaric-tag-based quantification data shown in Fig. 3, Extended Data Fig. 3 and Supplementary Tables 1 and 2 are available through PRIDE (ref. ^58^) with the identifiers PXD025366 and PXD030576. Label-free data shown in Fig. 3, Extended Data Fig. 5 and Table 3 are available through PRIDE (ref. ^58^) with the identifier PXD025369. Data for the gel band identification in Extended Data Fig. 6 and Supplementary Table 4 are available through PRIDE (ref. ^58^) with the identifier PXD025371. Source data are provided with this paper.

## Source data

Source Data Fig. 1
Source Data Fig. 2
Source Data Fig. 4
Source Data Fig. 5
Source Data Extended Data Fig. 1
Source Data Extended Data Fig. 2
Source Data Extended Data Fig. 3
Source Data Extended Data Fig. 6
Source Data Extended Data Fig. 7
