# Supplementary material for: Oocytes maintain ROS-free mitochondrial metabolism by suppressing complex I
Source: Nature. 2022 Jul 20;607(7920):756–61. doi: 10.1038/s41586-022-04979-5 (PMC9329100; doi:10.1038/s41586-022-04979-5)
Supplement: Supplementary file 1 — This file contains Supplementary Figs. 1 and 2 and legends for Supplementary Tables 1–4. [file 41586_2022_4979_MOESM1_ESM.pdf]

nature portfolio

https://doi.org/10.1038/s41586-022-04979-5

Supplementary information

Oocytes maintain ROS-free mitochondrial metabolism by suppressing complex I

In the format provided by the authors and unedited

Supplementary Information

Oocytes maintain ROS-free mitochondrial metabolism by suppressing complex I

Aida Rodríguez-Nuevo[1], Ariadna Torres-Sanchez[1], Juan M Duran[1], Cristian De Guirior[3,4,5], Maria Angeles Martínez-Zamora[3,4,5], Elvan Böke[1,2]

[1]Centre for Genomic Regulation (CRG), The Barcelona Institute of Science and Technology, Barcelona, Spain. [2]Universitat Pompeu Fabra (UPF), Barcelona, Spain. [3]Gynaecology Department, Institute Clinic of Gynaecology, Obstetrics and Neonatology, Hospital Clinic, Barcelona, Spain. [4]Faculty of Medicine, University of Barcelona, Barcelona, Spain. [5]Institut d'Investigacions Biomèdiques August Pi i Sunyer (IDIBAPS), Barcelona, Spain.

Correspondence: elvan.boke@crg.eu

Supplementary information guide:

Supplementary Table 1. The mitochondrial proteome of early and late-stage oocytes and muscle tissue from *Xenopus laevis*. The relative abundances of mitochondrial proteins found in *Xenopus* stage I and stage VI oocytes, and gastrocnemius muscle in three replicates.

Supplementary Table 2. The mitochondrial proteome of early oocytes, heart, liver and white adipose tissues from *Xenopus laevis*. The relative abundances of mitochondrial proteins found in *Xenopus* stage I oocytes, heart, liver and white adipose tissue (WAT) in two replicates.

Supplementary Table 3. The mitochondrial proteome of human primordial follicles and ovarian somatic cells. Abundances of mitochondrial proteins found in human primordial oocytes, and three different dilutions of ovarian somatic cells. Normalized abundances in columns I and J were used to generate Extended Figure 5.

Supplementary Table 4. Identified subunits of mitochondrial complex I and complex II in BN-PAGE gel-excised bands. List of complex I subunits detected in bands excised at ~1 MDa (sheet “~1 MDa”), or list of complex II subunits detected in bands excised at ~240 kDa (sheet “~240 kDa”) with the corresponding abundances, FDR confidence levels and number of unique peptides. Mitochondria were isolated from *Xenopus* stage I and stage VI oocytes, and gastrocnemius muscles (*X. laevis* and *M. musculus*).

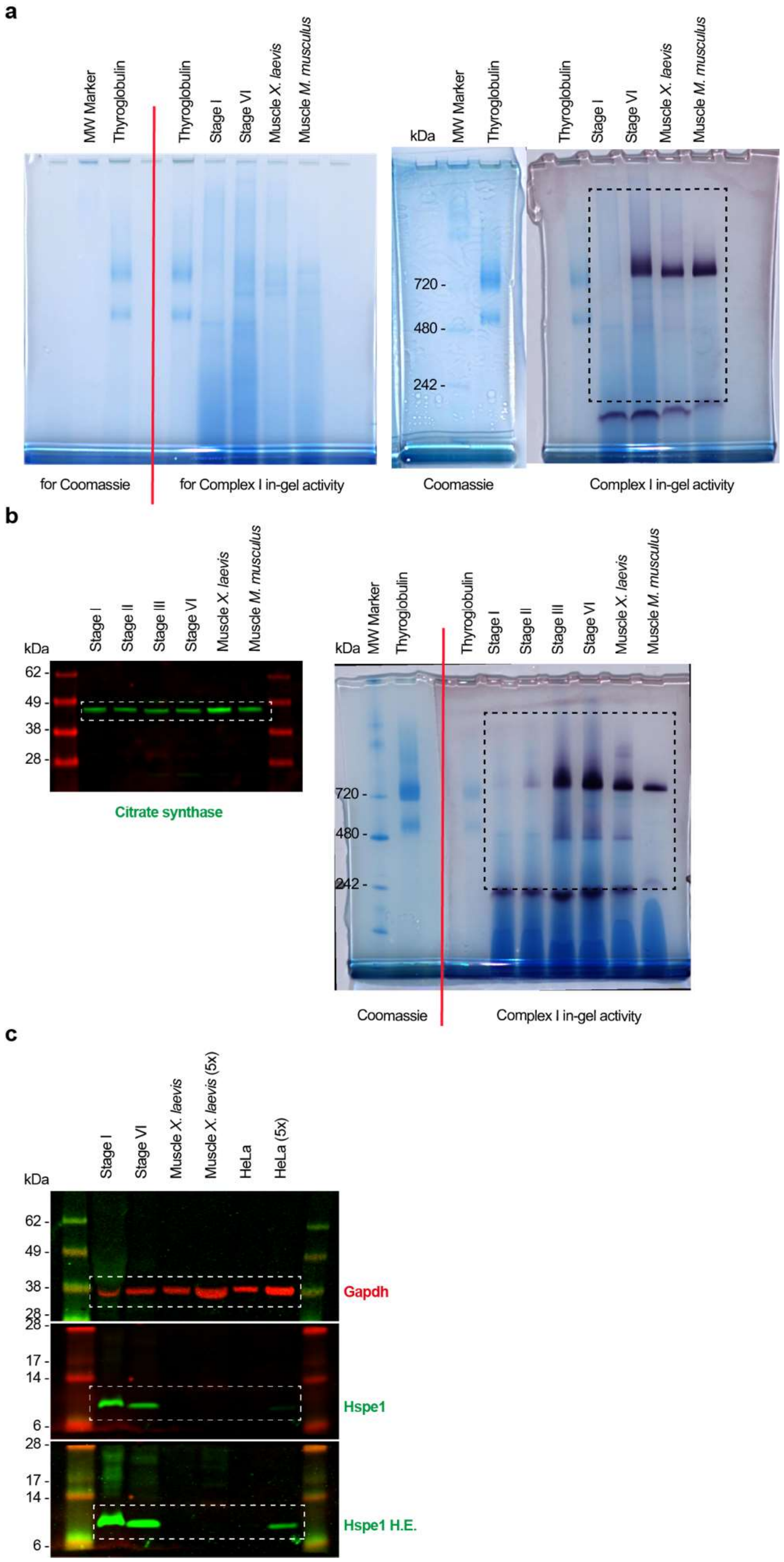

Supplementary Figure 1. Uncropped gels and immunoblots from main figures 4 and 5, and Extended Data figure 3. a, Shown in Fig 4a. Left panel: Entire gel after BN-

PAGE. Right panel: Gel assayed for complex I in-gel activity. Left part of the gel was cut and stained with Coomassie after BN-PAGE to reveal the native protein molecular weight (MW) marker. **b,** Left panel: For the BN-PAGE Shown in Fig. 5a. Samples were first run in SDS-PAGE gels and immunoblotted for Citrate synthase levels to ensure equal mitochondrial loading. Right panel: BN-PAGE gel assayed for complex I in-gel activity. Left part of the gel was cut and stained with Coomassie after BN-PAGE to reveal the native protein molecular weight (MW) marker. **c,** Shown in Extended Data Fig. 3j. SDS-PAGE immunoblot of Hspe1. Gapdh levels were assayed in the same blot and used as loading control.

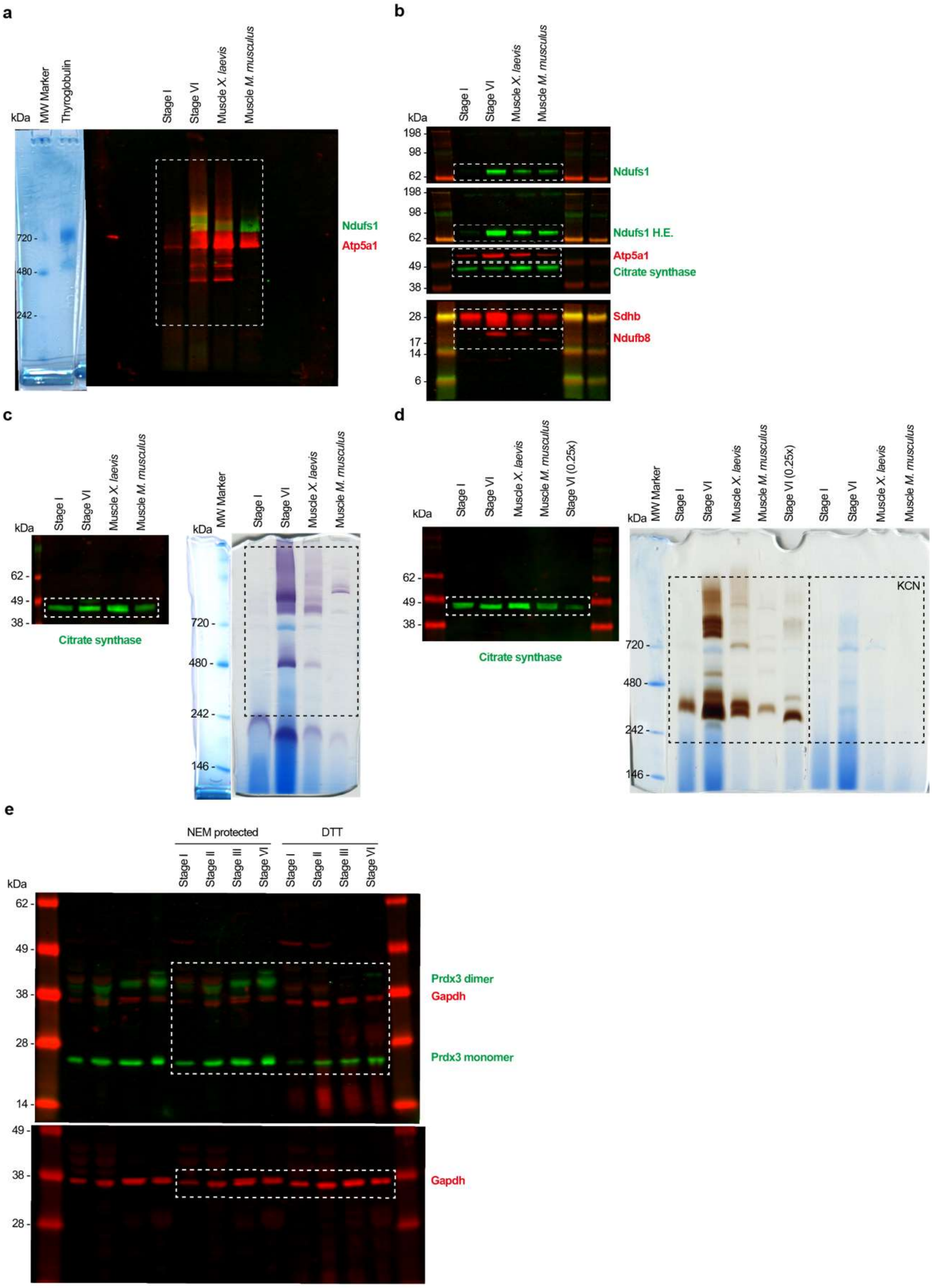

Supplementary Figure 2. Uncropped gels and immunoblots from Extended Data figures 6 and 7. a, Shown in Extended Data Fig 6c. Native immunoblot of Ndufs1 and Atp5a1. Left part of the gel was cut and stained with Coomassie after BN-PAGE to reveal the native protein molecular weight (MW) marker. **b,** Shown in Extended Data Fig 6d. SDS-PAGE immunoblots of Ndufs1, Atp5a1, SdhB and NdufB8 of same samples analysed in Fig. 4a. Citrate synthase levels were assayed in the same blot and used as

mitochondrial loading control. **c,** Left panel: For the BN-PAGE Shown in Fig. 6h. Samples were first run in SDS-PAGE gels and immunoblotted for Citrate synthase levels to ensure equal mitochondrial loading. Right panel: BN-PAGE gel assayed for complex I in-gel activity. Left part of the gel was cut and stained with Coomassie after BN-PAGE to reveal the native protein molecular weight (MW) marker. **d,** Left panel: For the BN-PAGE Shown in Fig. 6i. Samples were first run in SDS-PAGE gels and immunoblotted for Citrate synthase levels to ensure equal mitochondrial loading. Right panel: BN-PAGE gel assayed for complex IV in-gel activity in the absence or in the presence of KCN. Left part of the gel was cut and stained with Coomassie after BN-PAGE to reveal the native protein molecular weight (MW) marker. **e,** Shown in Extended Data Fig 7b. SDS-PAGE immunoblot of Prdx3 of samples either NEM protected or treated with DTT. Gapdh levels were assayed in the same blot and used as loading control.
