# Supplementary material for: Oocytes maintain ROS-free mitochondrial metabolism by suppressing complex I
Source: Nature. 2022 Jul 20;607(7920):756–61. doi: 10.1038/s41586-022-04979-5 (PMC9329100; doi:10.1038/s41586-022-04979-5)
Supplement: Supplementary file 2 — Reporting Summary [file 41586_2022_4979_MOESM2_ESM.pdf]

nature research

Corresponding author(s): Elvan Boke

Last updated by author(s): May 9, 2022

Reporting Summary

Nature Research wishes to improve the reproducibility of the work that we publish. This form provides structure for consistency and transparency in reporting. For further information on Nature Research policies, see our Editorial Policies and the Editorial Policy Checklist.

Statistics

For all statistical analyses, confirm that the following items are present in the figure legend, table legend, main text, or Methods section.

n/a	Confirmed	
☐	☒	The exact sample size (*n*) for each experimental group/condition, given as a discrete number and unit of measurement
☐	☒	A statement on whether measurements were taken from distinct samples or whether the same sample was measured repeatedly
☐	☒	The statistical test(s) used AND whether they are one- or two-sided<br>*Only common tests should be described solely by name; describe more complex techniques in the Methods section.*
☒	☐	A description of all covariates tested
☐	☒	A description of any assumptions or corrections, such as tests of normality and adjustment for multiple comparisons
☐	☒	A full description of the statistical parameters including central tendency (e.g. means) or other basic estimates (e.g. regression coefficient) AND variation (e.g. standard deviation) or associated estimates of uncertainty (e.g. confidence intervals)
☐	☒	For null hypothesis testing, the test statistic (e.g. *F*, *t*, *r*) with confidence intervals, effect sizes, degrees of freedom and *P* value noted<br>*Give P values as exact values whenever suitable.*
☒	☐	For Bayesian analysis, information on the choice of priors and Markov chain Monte Carlo settings
☒	☐	For hierarchical and complex designs, identification of the appropriate level for tests and full reporting of outcomes
☒	☐	Estimates of effect sizes (e.g. Cohen's *d*, Pearson's *r*), indicating how they were calculated

Our web collection on statistics for biologists contains articles on many of the points above.

Software and code

Policy information about availability of computer code

Data collection	Commercial software LAS X (Leica. v3.5.5.19976), Amersham Imager (GE Healthcare), Seahorse Wave (Agilent, v2.6), Tecan i-control (Tecan, v3.23), Image Studio (Li-COR, v5.2), and LightCycler (Roche, v1.5.1).
Data analysis	FIJI software (ImageJ 1.52d), Microsoft Excel (v16.16.6), GraphPad Prism (v6), Metabolon software, Proteome Discoverer software suite (v2.3, Thermo Fisher Scientific), Mascot search engine (v2.6, Matrix Science), and JMP (v13.2).

For manuscripts utilizing custom algorithms or software that are central to the research but not yet described in published literature, software must be made available to editors and reviewers. We strongly encourage code deposition in a community repository (e.g. GitHub). See the Nature Research guidelines for submitting code & software for further information.

Data

Policy information about availability of data

All manuscripts must include a data availability statement. This statement should provide the following information, where applicable:

- Accession codes, unique identifiers, or web links for publicly available datasets
- A list of figures that have associated raw data
- A description of any restrictions on data availability

Isobaric tag-based quantification data shown in Figure 3, Extended Data Figure 3, and Supplementary Tables 1 and 2 are available via PRIDE with identifiers PXD025366 and PXD030576. Label-free data shown in Figure 3, Extended Data Figure 5 and Table 3 are available via PRIDE with identifier PXD025369. Gel band identification shown in Extended Data Figure 6 and Table 4 are available via PRIDE with identifier PXD025371.

Field-specific reporting

Please select the one below that is the best fit for your research. If you are not sure, read the appropriate sections before making your selection.

☒ Life sciences ☐ Behavioural & social sciences ☐ Ecological, evolutionary & environmental sciences

For a reference copy of the document with all sections, see nature.com/documents/nr-reporting-summary-flat.pdf

Life sciences study design

All studies must disclose on these points even when the disclosure is negative.

Sample size	Sample sizes were chosen based on published studies (select examples listed below) to ensure reliable statistical testing and to account for variability among outbred populations. Experimental limitations were also taken into account, such as the number of primordial oocytes that could be obtained from human ovaries. Statistical tests were applied to evaluate the significance of studied effects for the given sample size.<br><br>https://doi.org/10.1016/j.cell.2016.06.051<br>https://doi.org/10.1016/j.cell.2015.07.017<br>https://doi.org/10.1038/ng.3844<br>https://doi.org/10.1093/humrep/dex284<br>https://doi.org/10.1038/s41586-021-03200-3<br>https://doi.org/10.1126/science.abj3944
Data exclusions	No data were excluded with the exception of few cases in which the full batch of oocytes died independently of the experimental condition used in the experiment.
Replication	The majority of the experiments were performed with at least 3 biological replicates involving outbred animals or human subjects. Sample limitation became too severe for human subjects during the coronavirus pandemic when elective surgeries are either canceled or severely reduced for long periods. Thus, in Figure 2a we only included two replicates from two individuals, but the data nevertheless had strong statistical power (p value = 0.0000121) as the effect is very reproducible between different oocytes and individuals. All experimental findings were reliably reproducible.
Randomization	All experiments were performed on isolated oocytes or tissues. Randomization was performed by two means. First, all outbred frogs used in this study were chosen by blinded animal facility personnel without our knowledge. Similarly, all human participants in the study was chosen by our collaborators in hospitals without our knowledge according to the inclusion criteria previously agreed. Second, all isolated oocytes or tissue samples were first grouped together and then randomly distributed to different experimental groups.
Blinding	Blinding during data collection was not required as standard experimental procedures were applied for different groups, such as western blots and immunohistochemistry. Blinding during data analysis was performed in oocyte survival experiments by involving multiple lab members for analysing blinded datasets. Blinding for the analysis of other experiments was not required since the different experimental groups were analysed using the same parameters.

Reporting for specific materials, systems and methods

We require information from authors about some types of materials, experimental systems and methods used in many studies. Here, indicate whether each material, system or method listed is relevant to your study. If you are not sure if a list item applies to your research, read the appropriate section before selecting a response.

Materials & experimental systems

n/a	Involved in the study	
☐	☒	Antibodies
☐	☒	Eukaryotic cell lines
☒	☐	Palaeontology and archaeology
☐	☒	Animals and other organisms
☐	☒	Human research participants
☒	☐	Clinical data
☒	☐	Dual use research of concern

Methods

n/a	Involved in the study	
☒	☐	ChIP-seq
☒	☐	Flow cytometry
☒	☐	MRI-based neuroimaging

Antibodies

Antibodies used	1. ATP5A1 (Abcam; Catalog #ab14748, Clone 15H4C4, Lot GR3306993-21)<br>2. Citrate synthase (Abcam; Catalog #ab96600, Lot GR3362557-2)<br>3. GAPDH (Thermo; Catalog #AM4300, Clone 6C5, Lot 00959879)<br>4. HSPE1 (Thermo, Catalog #PA5-30428, Lot VJ3112392C)<br>5. NDUFB8 (Abcam; Catalog #ab110242, Clone 20E9DH10C12 Lot GR3264924-7)

6. NDUFS1 (Abcam; Catalog #ab169540, Clone EPR11521(B) Lot GR124081-13)
7. PRDX3 (Abcam; Catalog #ab73349, Lot GR3283254-1)
8. SDHB (Abcam; Catalog #ab14714, Clone 21A11AE7 Lot GR3272683-2)
9. anti-mouse IgG DyLight 680 (Thermo, #35518, LOTVG299844)
10. anti-rabbit IgG DyLight 800 4X PEG (Thermo, #SA5-35571, Lot SE251154B)
11.anti-rabbit Alexa488 (Thermo, #A-11008, Lot 2256822)
12. anti-rabbit Alexa555 (Thermo, #A-21428, Lot 2308257)
13. anti-mouse Alexa647 (Thermo, #A-21236, Lot 2326487)

Validation

We have only purchased and used antibodies from credible suppliers (Abcam or Thermo) with ample proof of validation in the literature. Of course, all experiments were performed with negative (no primary antibody) and positive (He-La cells/extracts) controls. Below is a detailed list of antibodies used in this study, we include two examples each (among tens or hundreds available in the literature) for validation. Antibodies against:

ATP5A1:
Ref1: https://doi.org/10.1038/nature19754
Ref2: https://doi.org/10.1016/j.molcel.2020.12.034

Citrate synthase:
Ref1: https://doi.org/10.15252/embj.2019102817
Ref2: https://doi.org/10.1038/s41598-019-42902-7

GADPH has been widely used as a loading control in the literature:
Ref1: https://doi.org/10.1038/s41467-019-11671-2
Ref2: https://doi.org/10.1038/nature14156

HSPE1 was chosen because of the manufacturers prediction to reactivity to all Xenopus, mouse and human proteins; and was validated in our lab before for mitochondrial localisation.

NDUFB8:
Ref1: https://doi.org/10.1073/pnas.2000640117
Ref2: https://doi.org/10.1007/s00401-017-1794-7

NDUFS1:
Ref1: https://doi.org/10.1016/j.cmet.2017.03.010
Ref2: https://doi.org/10.1172/JCI79964

PRDX3:
Ref1: https://doi.org/10.1016/j.freeradbiomed.2020.06.020
Ref2: https://doi.org/10.1016/j.chembiol.2018.12.002
This antibody was validated by the supplier in genetic knockout of endogenous PRDX3 in HAP1 cells.

SDHB:
Ref1: https://doi.org/10.1038/s41467-021-22117-z
Ref2: https://doi.org/10.1073/pnas.2000640117
This antibody was validated by the supplier in genetic knockout of endogenous SDHB HEK293 cells.

anti-mouse IgG DyLight 680:
Ref1: https://doi.org/10.1038/s42255-021-00366-y
Ref2: https://doi.org/10.3389/fcell.2021.683038
The specificity of the antibody was validated by the supplier checking the lack of fluorescence in the absence of mouse IgG antibodies.

anti-rabbit IgG DyLight 800 4X PEG:
Ref1: https://doi.org/10.1016/j.jmb.2021.167277
Ref2: https://doi.org/10.1083/jcb.202012114
The specificity of the antibody was validated by us checking the lack of fluorescence in the absence of rabbit IgG antibodies.

anti-rabbit Alexa488:
Ref1: https://doi.org/10.1007/s00709-021-01678-2
Ref2: https://doi.org/10.1016/j.redox.2022.102280
The specificity of the antibody was validated by the supplier checking the lack of fluorescence in the absence of rabbit IgG antibodies.

anti-rabbit Alexa555:
Ref1: https://doi.org/10.1016/j.jbc.2022.101904
Ref2: https://doi.org/10.1242/dev.200200
The specificity of the antibody was validated by the supplier checking the lack of fluorescence in the absence of rabbit IgG antibodies.

anti-mouse Alexa647
Ref1: https://doi.org/10.1038/s41467-022-29967-1
Ref2: https://doi.org/10.3390/molecules27082440
The specificity of the antibody was validated by the supplier checking the lack of fluorescence in the absence of mouse IgG antibodies.

Eukaryotic cell lines

Policy information about cell lines

Cell line source(s)	Hela cells were obtained from ATCC (CCL2).
Authentication	Cells were evaluated based on morphological inspection.
Mycoplasma contamination	Cells were negative for mycoplasma contamination in tests performed regularly.
Commonly misidentified lines (See ICLAC register)	No commonly misidentified cell lines were used in this study.

Animals and other organisms

Policy information about studies involving animals; ARRIVE guidelines recommended for reporting animal research

Laboratory animals	Xenopus laevis adult females between 2 and 4 years old were purchased from Nasco (NJ, USA) and maintained in water tanks in the following controlled conditions: 18-21ºC, pH 6.8-7.5, O2 4-20 ppm, conductivity 500-1500 µs, ammonia < 0.1 ppm.<br>The C57BL/6J mice used in the experiments were purchased from Charles River laboratories and maintained in the Animal Facility of the Barcelona Biomedical Research Park (PRBB, Barcelona, Spain, EU) under specific pathogen-free conditions at 22°C with 40-60% humidity, in a 12 hours light/dark cycle, and with access to food and water ad libitum. Female mice of 7 weeks-of-age were used for extracting muscle tissue.
Wild animals	The study did not involve wild animals
Field-collected samples	The study did not involve samples collected from the field.
Ethics oversight	Animals used in this study were housed in the Barcelona Biomedical Research Park, accredited by the International Association for Assessment and Accreditation of Laboratory Animal Care (AAALAC). All use of animals was performed according to the requirements of directive 2010/63 EU and the Guide. All animals mentioned in the manuscript were sacrificed by accredited animal facility personnel before their tissues were extracted.

Note that full information on the approval of the study protocol must also be provided in the manuscript.

Human research participants

Policy information about studies involving human research participants

Population characteristics	Women aged from 18 to 35 undergoing ovarian surgery.
Recruitment	Women fulfilling the inclusion criteria undergoing ovarian surgery were asked to participate in the study. No other biases are present. Informed consent was obtained from all women.<br>Inclusion criteria: Age between 18 to 35, fertile (assessed by un-induced menstrual cycles or presence of antral follicles identified by ultrasound examination), presence of at least one ovary, signed informed consent.<br>Exclusion criteria: women with menopause, endometriosis, or who underwent bilateral oophorectomy.
Ethics oversight	Ethical Committee permission to work with primordial oocytes from human ovary samples was obtained from the Comité Ètic d'Investigació Clínica CEIC-Parc de salut MAR (Barcelona) and Comité Ético de investigación clínica CEIC-Hospital Clínic de Barcelona with approval number HCB/2018/0497. Written informed consent was obtained from all participants prior to their inclusions in the study.

Note that full information on the approval of the study protocol must also be provided in the manuscript.
